# *Debaryomyces hansenii* Strains Isolated From Danish Cheese Brines Act as Biocontrol Agents to Inhibit Germination and Growth of Contaminating Molds

**DOI:** 10.3389/fmicb.2021.662785

**Published:** 2021-06-15

**Authors:** Chuchu Huang, Ling Zhang, Pernille Greve Johansen, Mikael Agerlin Petersen, Nils Arneborg, Lene Jespersen

**Affiliations:** Department of Food Science, Faculty of Science, University of Copenhagen, Copenhagen, Denmark

**Keywords:** *Debaryomyces hansenii*, antagonistic activities, biocontrol, contaminating molds, cheese brine

## Abstract

The antagonistic activities of native *Debaryomyces hansenii* strains isolated from Danish cheese brines were evaluated against contaminating molds in the dairy industry. Determination of chromosome polymorphism by use of pulsed-field gel electrophoresis (PFGE) revealed a huge genetic heterogeneity among the *D. hansenii* strains, which was reflected in intra-species variation at the phenotypic level. 11 *D. hansenii* strains were tested for their ability to inhibit germination and growth of contaminating molds, frequently occurring at Danish dairies, i.e., *Cladosporium inversicolor, Cladosporium sinuosum, Fusarium avenaceum, Mucor racemosus*, and *Penicillium roqueforti.* Especially the germination of *C. inversicolor* and *P. roqueforti* was significantly inhibited by cell-free supernatants of all *D. hansenii* strains. The underlying factors behind the inhibitory effects of the *D. hansenii* cell-free supernatants were investigated. Based on dynamic headspace sampling followed by gas chromatography-mass spectrometry (DHS-GC-MS), 71 volatile compounds (VOCs) produced by the *D. hansenii* strains were identified, including 6 acids, 22 alcohols, 15 aldehydes, 3 benzene derivatives, 8 esters, 3 heterocyclic compounds, 12 ketones, and 2 phenols. Among the 71 identified VOCs, inhibition of germination of *C. inversicolor* correlated strongly with three VOCs, i.e., 3-methylbutanoic acid, 2-pentanone as well as acetic acid. For *P. roqueforti*, two VOCs correlated with inhibition of germination, i.e., acetone and 2-phenylethanol, of which the latter also correlated strongly with inhibition of mycelium growth. Low half-maximal inhibitory concentrations (IC_50_) were especially observed for 3-methylbutanoic acid, i.e., 6.32–9.53 × 10^–5^ and 2.00–2.67 × 10^–4^ mol/L for *C. inversicolor* and *P. roqueforti*, respectively. For 2-phenylethanol, a well-known quorum sensing molecule, the IC_50_ was 1.99–7.49 × 10^–3^ and 1.73–3.45 × 10^–3^ mol/L for *C. inversicolor* and *P. roqueforti*, respectively. For acetic acid, the IC_50_ was 1.35–2.47 × 10^–3^ and 1.19–2.80 × 10^–3^ mol/L for *C. inversicolor* and *P. roqueforti*, respectively. Finally, relative weak inhibition was observed for 2-pentanone and acetone. The current study shows that native strains of *D. hansenii* isolated from Danish brines have antagonistic effects against specific contaminating molds and points to the development of *D. hansenii* strains as bioprotective cultures, targeting cheese brines and cheese surfaces.

## Introduction

Mold contamination is a major problem occurring in food processing, which not only leads to severe economic losses and food waste but also may influence food safety (Garnier et al., 2017). In the dairy industry, mold contamination can appear throughout the entire cheese production as cheeses come into contact with processing equipment and air. Mold growth is especially seen during cheese ripening, storage, and distribution which results in a reduction of cheese quality including visible and invisible defects such as mold colonization, off-flavors, and potential risk of mycotoxin formation (Kure and Skaar, 2019). As a consequence, antifungal compounds and potential biocontrol agents have received increasing interest to prevent the growth of contaminating molds on cheeses (Irlinger and Mounier, 2009).

Yeasts are considered relevant for biocontrol applications, due to their simple cultivation requirements and limited biosafety concerns (Freimoser et al., 2019). A number of yeast species have been proven to have antagonistic activities against molds, though mostly applied in the control of post-harvest diseases of fruits (Bencheqroun et al., 2007; Liu et al., 2013; Grzegorczyk et al., 2017; Czarnecka et al., 2019). Within food production, antagonistic yeasts have been reported on, e.g., meat and dairy products (Liu and Tsao, 2010; Andrade et al., 2014) but their application is lacking behind. A number of antifungal volatile organic compounds (VOCs) produced by biocontrol yeasts have been associated with fungal inhibition, i.e., several alcohols (2-phenylethanol, ethanol, 2-methyl-1-butanol and 3-methyl-1-butanol, 2-methyl-1-propanol, and isoamyl alcohol) (Fialho et al., 2010; Ando et al., 2012; Núñez et al., 2015; Contarino et al., 2019) and esters (ethyl acetate, isoamyl acetate, phenylethyl acetate, isobutyl acetate, 2-phenyl ethyl acetate, and ethyl propionate) (Masoud et al., 2005; Fialho et al., 2010; Choińska et al., 2020). Although several studies state the production of VOCs involved in the antifungal activities of biocontrol yeasts, only a few of the aforementioned single VOCs have been investigated in depth. Also other antifungal actions of antagonistic yeasts have been reported including the competition for space and nutrients between yeasts and molds (Andrade et al., 2014; Medina-Córdova et al., 2016) as well as killer toxins playing a role in the defense system of yeasts against molds (Santos and Marquina, 2004; Grzegorczyk et al., 2017).

A number of yeasts have been detected and isolated from dairy products and dairy environment (Fröhlich-Wyder et al., 2019). Among these, *D. hansenii* has qualified presumption of safety (QPS) status by the European Food Safety Authority (EFSA) (Koutsoumanis et al., 2020) making it suitable as a biocontrol yeast in dairy products. *D. hansenii* is a halophilic yeasts being dominating among yeast species associated with most cheese varieties (Gori et al., 2012). Previously, we found that *D. hansenii* is by far the most predominant yeast species isolated from Danbo cheese brines reaching ≥ 3.5 log_10_ CFU/mL (Haastrup et al., 2018). Moreover, *D. hansenii* is a highly heterogeneous species showing phenotypic differences at the strain level. Variations among strains include differences in the ability to assimilate and ferment different carbon sources, secretion of dissimilar lipases and proteases, and diverse preferable growth conditions (Petersen and Jespersen, 2004). Consequently, strain variations in antagonistic potential might be expected. Antifungal activities of *D. hansenii* against contaminating molds are reported for several foods including dry-cured meat products (Andrade et al., 2014; Núñez et al., 2015), fruits (Hernández-Montiel et al., 2010), and dairy products (Van Den Tempel and Jakobsen, 2000; Liu and Tsao, 2009; Lessard et al., 2012). In dairy products, *D. hansenii* is reported to reduce the growth of *Penicillium camemberti* (Lessard et al., 2012). Further, *D. hansenii* strains obtained from blue mold cheeses are able to weakly inhibit *P. roqueforti* under aerobic conditions (Van Den Tempel and Jakobsen, 2000). Up to now, several factors have been found to influence the antifungal efficiency of *D. hansenii*, including water activity (a_w_), temperature, nutrient availability (Andrade et al., 2014; Núñez et al., 2015), and the concentration of molds (Liu and Tsao, 2009). Further, the production of metabolites varies among *D. hansenii* strains (Núñez et al., 2015; Grzegorczyk et al., 2017; Hernandez-Montiel et al., 2018). In previous studies, *D. hansenii* strains have been shown to exhibit varying antifungal actions (Van Den Tempel and Jakobsen, 2000; Medina-Córdova et al., 2018), however, further in-depth studies are required to explore these actions. We have previously determined the distinctive growth characteristics and NaCl tolerance of *D. hansenii* strains isolated from Danish cheese brines (Zhang et al., 2020), however, their potential abilities to inhibit mold contaminants from dairy environments have not, as yet, been explored.

This paper aims to evaluate the antifungal activities of *D. hansenii* strains isolated from Danish cheese brines against different contaminating molds. For this purpose, the effects of different *D. hansenii* strains were examined for their inhibitory capacity on germination and growth of contaminating molds including determination of the half-maximal inhibitory concentration (IC_50_) of single VOCs.

## Materials and Methods

### Yeast and Mold Strains, Media, and Growth Conditions

All *D. hansenii* strains used in this study were previously isolated and identified by Haastrup et al. (2018) from three different Danish dairies; *D. hansenii* strains KU-9, KU-10, KU-11, and KU-12 were isolated from dairy A (vat1); KU-27, KU-28, KU-29, and KU-30 were isolated from dairy A (vat2); KU-72 was isolated from dairy B; KU-78 and KU-80 were isolated from dairy C (vat1 and vat2, respectively). *C. inversicolor*, *C. sinuosum*, and *F. avenaceum* were obtained from a Danish dairy and their identification was verified by Deutsche Sammlung von Mikroorganismen (DSM, Germany). *M. racemosus* DSM5266 (isolated from cheese) and *P. roqueforti* DSM1079 (isolated from cheese) were obtained from DSM. All molds were capable of growing on cheese agar, prepared according to Sørensen et al. (2011) at 25°C for 7 days (Supplementary Figure 1). Of these, *C. inversicolor*, *C. sinuosum*, *F. avenaceum*, and *P. roqueforti* reproduce asexually by the formation of conidia, while *M. racemosus* reproduce sexually by the formation of spores. In the following, spores will be used when referring to these reproductive cells of the five molds.

Yeast strains were propagated at 25°C in Malt Yeast Glucose Peptone broth added 4% (w/v) NaCl (MYGP added 4% (w/v) NaCl; per liter, 10 g D (+)-glucose monohydrate (Merck, Darmstadt, Germany), 5 g bactopeptone (BD, Detroit, MI, United States), 3 g yeast extract (BD), 3 g malt extract (BD), 40 g NaCl (Merck) with pH adjusted to 5.3 ± 0.1) or MYGP agar added 4% (w/v) NaCl (adding 20 g bacto agar (BD) to MYGP broth added 4% (w/v) NaCl). Mold species were routinely grown at 25°C in Malt Extract medium (MEB; per liter; 20 g malt extract (BD), 10 g D(+)-glucose monohydrate (Merck), 5 g bactopeptone (BD) with pH adjusted to 5.3 ± 0.1) or on malt extract agar (MEA, adding 20 g bacto agar (BD) to MEB broth components).

### Preparation of Spores

Molds were cultured on MEA at 25°C for 7-14 days. Spores were suspended in saline peptone (SPO) solution (per liter; 5 g NaCl (Merck), 0.3 g Na_2_HPO_4_⋅2H_2_O (Merck), 1 g bactopeptone (BD) with pH adjusted to 5.3 ± 0.1) containing 0.01% Tween 80 (Sigma-Aldrich, St. Louis, United States) followed by filtering through six layers of sterilized gauze to remove hyphal fragments. Spore concentrations were estimated by bright field microscopy (Olympus BX40, Japan) using a Neubauer counting chamber and adjusted to a final concentration of 10^4^ spores/mL using SPO solution.

### Pulsed-Field Gel Electrophoresis and Cluster Analysis of *D. hansenii* Strains

The DNA preparation of yeast strains and the running condition of pulsed-field gel electrophoresis (PFGE) were performed according to Petersen and Jespersen (2004). The gels were visualized with UV transillumination and photographed (alphaeasefc software, Alpha-InnoTec GmbH, Germany). Estimation of the band size was analyzed using the LabImage 1D, ver.7.1.3 software (Kapelan Bio-Imaging, Germany). The cluster analysis was carried out using BioNumerics version 7.1 (Applied Maths, Kortrijk, Belgium). The similarities between profiles of bands were determined using the fraction of shared bands (Dice coefficient), and the cluster analysis was calculated by the unweighted pair group method using arithmetic average linkage (UPGMA method).

### Change in pH and Cell Counts of *D. hansenii*

All *D. hansenii* strains were grown in MYGP broth added 4% (w/v) NaCl overnight. OD_600__nm_ of the overnight cultures were measured using a spectrophotometer (UV 1800, Shimadzu, Japan) followed by dilution with fresh media at initial concentration of 0.01 ± 0.005 (OD_600nm_). The cell cultures were grown in MYGP broth added 4% (w/v) NaCl for 72 h at 25°C. Measurements of pH were carried out using an electrode (In Lab 426, Mettler-Toledo, Glostrup, Denmark) connected to a pH meter (1120, Mettler-Toledo) and plate counting method on MYGP agar added 4% (w/v) NaCl was used to monitor the growth of *D. hansenii* at the initial and end time point.

### Effect of *D. hansenii* Cell-Free Supernatants on the Growth of Molds

Cell-free supernatants were made for each *D. hansenii* strain in the stationary growth phase (cultured in MYGP broth added 4% (w/v) NaCl for 72 h at 25°C) by centrifugation (3000 × *g*, 10 min) followed by filtering the supernatants using 0.22 μm pore size filter (Frisenette ApS, Knebel, Denmark).

Aliquots of 100 μL of double-strength MEB containing 10^4^ spores/mL plus 100 μL of the cell-free supernatant collected from each *D. hansenii* strains or 100 μL of Yeast Peptone (per liter, 5 g bactopeptone, 3.0 g yeast extract, 40 g NaCl, pH 5.3) as control were loaded into wells (96 microtiter plates, Corning, New York, America). The antifungal activities of cell-free supernatants against molds were determined by measuring the growth curve using oCelloScope^TM^ Unisensor (Philips BioCell A/S, Denmark) at 25°C for 48 h. The oCelloScope detection system (objective, 4×) was described in detail by Fredborg et al. (2013). The image distance was 4.90 μm and the illumination exposure time was 2 ms. Time-lapse scanning microscopy through a fluid sample was conducted thereby generating a series of 6 images in each well, every 2 h. Growth curves were generated automatically by using the segmentation and extraction of surface areas (SESA) algorithm in UniExplorer (Philips BioCell A/S, Denmark).

Germination ratios of spores were analyzed by ImageJ (v1.51g-v1.51n; Fiji package). A spore with a germination tube longer than the spore itself was considered as a germinated spore. For each image, 90-120 spores were counted and relative germination ratios were calculated as follows:

$$\frac{g_{1}}{t_{1}}/\frac{g_{0}}{t_{0}}\times 100\%$$

where g_1_ is the number of germinated spores added cell-free supernatant, t_1_ is the total number of spores added cell-free supernatant, g_0_ is the number of germinated spores without cell-free supernatant, t_0_ is the total number of spores without cell-free supernatant. The time points for counting germination ratio were chosen according to the method from Trinci (1971).

Growth rate (μ) values were analyzed using the DMfit software available on the Combase website^1^ based on the aforementioned growth curve values and the model proposed by Baranyi and Roberts (1994). Relative mycelium growth rate was calculated as follows:

$$\frac{\mu_{1}}{\mu_{0}}\times 100\%$$

where μ_1_ is the growth rate added cell-free supernatant, μ_0_ is the growth rate without yeast cell-free supernatant. The μ values fit with *R*^2^ > 0.9 were considered valid data.

### Determination of Volatile Compounds (VOCs) Based on GC–MS Spectrometry

*Debaryomyces hansenii* cell-free supernatants were prepared as described above. VOCs of supernatants were collected in a dynamic headspace sampling (DHS) system. Each sample contained 20 mL supernatant sample plus 1 mL 4-methyl-1-pentanol (5 ppm) as the internal standard. The gas flask equipped with a purge head was equilibrated at 37°C in a water bath with magnetic stirring (200 rpm), and then purged with nitrogen (100 mL/min, 20 min). VOCs were collected by Tenax-TA traps (250 mg, mesh size 60/80, Buchem BV, Apeldoorn, Netherlands). Then the traps were continually purged with nitrogen (100 mL/min, 10 min) to remove excess water. The dynamic headspace collection was carried out in duplicates for all samples. VOCs were analyzed using gas chromatography-mass spectrometry (GC–MS) as described by Liu et al. (2015) and were identified based on the commercial database (Wiley275.L, HP product no. G1035A). The identified compounds were confirmed by comparing with retention indices (RI) of authentic reference compounds or the average of retention indices reported in the literature. All identified VOCs were semi-quantified as peak areas in the total ion chromatogram (TIC).

### Evaluation of Inhibitory Effects of Single Targeted VOCs on Molds

Volatile compounds were selected based on correlation between the relative peak area of each compound produced by *D. hansenii* and the inhibition data (relative germination ratio and relative mycelium growth rate obtained from the experiments described in the section “Effect of *D. hansenii* Cell-Free Supernatants on the Growth of Mold**s**”) through Spearman correlation analysis. Those with significant strong correlations (*P* < 0.05) were selected for the subsequent experiments and purchased from Sigma-Aldrich. The interpretation of the rank correlation was according to the rules in Akoglu (2018).

Aliquots of 200 μL of MEB containing 10^4^ spores/mL supplemented with acetic acid, 3-methylbutanoic acid, acetone, 2-pentanone, and 2-phenylethanol (Sigma-Aldrich, St. Louis, MO, United States) to the final concentrations 10^0^, 10^–1^, 10^–2^, 10^–3^, 10^–4^, 10^–5^, and 10^–6^ mol/L were loaded into 96 well plates. Aliquots of 200 μL of MEB containing 10^4^ spores/mL were included as controls. The antifungal activities of single identified VOCs were determined by measuring the growth curve using oCelloScope^TM^ Unisensor at 25°C for 48 h. Relative germination ratios and relative mycelium growth rates were calculated as described above. In addition, IC_50_ of single targeted VOCs for each mold was calculated. Long-term impacts of the five single targeted VOCs on the formation of mycelial pellicles were evaluated by prolonged incubation of the 96-well microtiter plates at 25°C for 7 days with the concentrations of the five single targeted VOCs as listed above. By visual inspection inhibition of mycelial pellicle formation was scored.

### Quantification of Inhibitory VOCs Produced by *D. hansenii* Strains

Standard curves based on matrix-spiked samples were performed for two acids (acetic acid and 3-methylbutanoic acid), one alcohol (2-phenylethanol), and two ketones (acetone, and 2-pentanone). The peak area of the quantifier ion was used for estimating the concentration of each aforementioned VOCs produced by *D. hansenii* strains.

### Data Analysis

Data were expressed as mean ± standard deviation and compared using one-way ANOVA analyzed by SPSS 17.0. Comparison of means was performed using Duncan’s multiple range test and the statistical significance was applied at the level *P* < 0.05. IC_50_ of single VOCs was analyzed by GraphPad Prism 5, while the Spearman correlation analysis was analyzed by R software (“Hmisc” package).

## Results

### *Debaryomyces hansenii* Strains Isolated From Cheese Brines Had Huge Genetic Diversity

**FIGURE 1:**
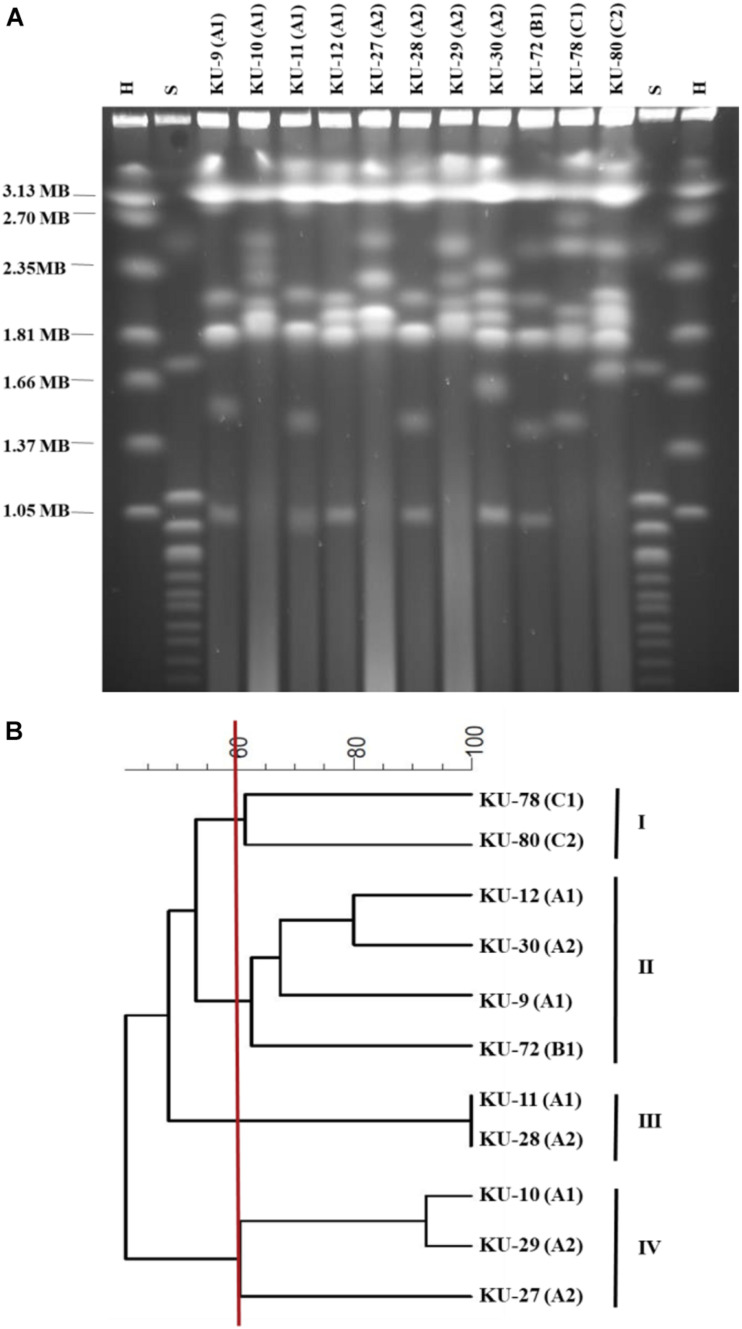
**(A)** Chromosome polymorphism of *D. hansenii* strains isolated from Danish cheese brines. Pulsed-field gel electrophoresis (PFGE) was carried out at a 200 switch interval at 150 V for 24 h followed by a 700 s switch interval at 100 V for 48 h. Markers: H, *Hansenula wingei*; S, *Saccharomyces cerevisiae*. **(B)** Dice/UPMGA clustering dendrogram of *D. hansenii* strains.

The chromosomal profiles of the 11 *D. hansenii* strains isolated from brines at three different Danish dairies are shown in Figure 1 and bands sizes are reported in Supplementary Table 1. Among the strains, genetic diversity was evident. The numbers of chromosomal bands varied between five and seven with sizes from 0.98 to 3.14 Mb. A cluster analysis based on the chromosomal profiles divided the 11 *D. hansenii* strains into four clusters at a similarity level of 60% (Figure 1B). Nine of the *D. hansenii* strains had unique profiles, while the two strains in cluster III (KU-11 and KU-28) isolated from two different vats at dairy A (A1 and A2) had identical profiles. *D. hansenii* strains (KU-9, KU-10, KU-11, KU-12, KU-27, KU-28, KU-29, and KU-30) isolated from the two vats at dairy A (A1 and A2) were divided into three different clusters (cluster II, III, and IV). Further, strains from dairy C were in cluster I (KU-78 and KU-80), clearly separated from the remaining dairies. On the contrary, no clear separation could be observed between dairy A and B as they clustered together in cluster II (KU-9, KU-12, KU-30, and KU-72), though at low similarity.

### Strain Variations in Acidification and Deacidification Abilities of *D. hansenii* Were Observed

Growth and changes in pH of *D. hansenii* strains grown in MYGP added 4% (w/v) NaCl for 72 h at 25°C are shown in Table 1. Examples of growth curves of *D. hansenii* strains are shown in Supplementary Figure 2. The initial inocula of the 11 *D. hansenii* strains were 0.7 ± 0.2 × 10^4^ CFU/mL and reached 4.2 ± 1.7 × 10^7^ CFU/mL after 72 h of growth. Even though all of the 11 *D. hansenii* strains grew to similar levels there were huge variations in their acidification abilities. After 72 h growth, acidification of MYGP from pH 5.3 to a minimum of 4.3 was observed for six *D. hansenii* strains (KU-9, KU-11, KU-12, KU-28, KU-30, and KU-72). Contrary, deacidification of MYGP from pH 5.3 to a maximum of 6.1 was observed for four *D. hansenii* strains (KU-10, KU-27, KU-29, and KU-78), whereas one strain (KU-80) did not significantly change the pH during the 72 h growth. Hence, compared to the cluster analysis, the genetic diversity and acidification/deacidification abilities were partly correlated. The acidifying strains were in clusters II and III, while cluster IV comprised three of the four deacidifying strains. Finally, cluster I contained one deacidifying strain (KU-78) and one strain that did not change pH (KU-80), however, rather high genetic differences between the two strains were detected at 62%.

**TABLE 1:** Growth of *D. hansenii* strains and change in pH of MYGP broth added 4% (w/v) NaCl after 72h inoculation at 25°C.

PFGE Cluster	Strain	Growth (CFU/mL) after 72h	pH after 72 h
I	KU-78	3.9 × 10^7^ ± 0.5 × 10^7a^	6.1 ± 0.0^f^
I	KU-80	8.3 × 10^7^ ± 0.5 × 10^7c^	5.2 ± 0.1^d^
II	KU-12	3.5 × 10^7^ ± 0.9 × 10^7a^	4.5 ± 0.0^b^
II	KU-30	2.7 × 10^7^ ± 0.7 × 10^7a^	4.8 ± 0.0^c^
II	KU-9	4.0 × 10^7^ ± 0.2 × 10^7a^	4.3 ± 0.2^a^
II	KU-72	3.4 × 10^7^ ± 0.4 × 10^7a^	4.5 ± 0.1^b^
III	KU-11	3.7 × 10^7^ ± 0.6 × 10^7a^	4.5 ± 0.1^b^
III	KU-28	2.6 × 10^7^ ± 0.5 × 10^7a^	4.6 ± 0.1^ab^
IV	KU-10	3.9 × 10^7^ ± 0.7 × 10^7a^	6.1 ± 0.1^f^
IV	KU-29	4.3 × 10^7^ ± 0.6 × 10^7a^	5.9 ± 0.1^e^
IV	KU-27	6.2 × 10^7^ ± 0.6 × 10^7b^	6.1 ± 0.1^f^

*Values in each line marked by different lowercase letters are significantly different using one-way ANOVA with Duncan’s test (≥95% confidence, *n* = 3). Clusters are based on chromosome polymorphism between the stains as determined by PFGE.*

### *Debaryomyces hansenii* Cell-Free Supernatants Affected Germination and Mycelium Growth of Contaminating Molds in a Mold Species Dependent Manner

Effects on germination ratios and mycelium growth rates of the five molds when exposed to *D. hansenii* cell-free supernatants collected from stationary phase cultures are shown in Table 2. For the germination ratios, the highest significant (*P <* 0.05) inhibition, among the five molds, was observed for *C. inversicolor*, with germination ratios below 5% when exposed to cell-free supernatants of all *D. hansenii* strains. For *P. roqueforti*, cell-free supernatants of *D. hansenii* KU-9 and KU-11 showed significant strong inhibition on the germination ratio to 9.5% and 6.8%, respectively, compared to cell-free supernatants of the other *D. hansenii* strains (13.7-33.8%). Slight inhibition by *D. hansenii* cell-free supernatants was observed on the germination ratio of *C. sinuosum* (82.9%). Contrary, no significant (*P* > 0.05) inhibitory effect on the germination ratios of *F. avenaceum* and *M. racemosus* was observed for any of the *D. hansenii* cell-free supernatants.

**TABLE 2:** Germination ratios (%) and mycelium growth rates (μ/h) of molds in presence of *D. hansenii* cell-free supernatants as compared to the control.

		*D. hansenii* strains											
PFGE cluster		I	I	II	II	II	II	III	III	IV	IV	IV	
		
Mold growth	Mold species	KU-78	KU-80	KU-12	KU-30	KU-9	KU-72	KU-11	KU-28	KU-10	KU-29	KU-27	Average
Germination ratio	*C. inversicolor*	0.4 ± 0.4^a^	0.0 ± 0.0	0.8 ± 0.4^ab^	0.8 ± 0.8^ab^	4.5 ± 0.6^d^	0.4 ± 0.4^a^	1.9 ± 0.4^bc^	1.9 ± 1.1^bc^	1.4 ± 0.9^abc^	1.1 ± 0.0^ab^	0.4 ± 0.4^a^	1.2 ± 1.5
	*C. sinuosum*	74.6 ± 6.6^ab^	91.6 ± 12.6^bcd^	83.6 ± 6.5^abc^	98.3 ± 18.3^bcd^	82.0 ± 26.0^abc^	86.7 ± 2.2^bc^	74.6 ± 11.1^ab^	88.7 ± 1.1^bc^	67.3 ± 11.0^a^	86.9 ± 5.9^bc^	77.9 ± 11.0^ab^	82.9 ± 19.6
	*F. avenaceum*	102.6 ± 2.1^ab^	96.1 ± 3.6^a^	99.6 ± 0.5^ab^	97.3 ± 4.1^ab^	98.8 ± 4.4^ab^	98.4 ± 2.3^ab^	99.4 ± 4.0^ab^	100.6 ± 2.8^ab^	102.7 ± 1.1^b^	99.3 ± 2.8^ab^	97.5 ± 2.6^ab^	99.3 ± 4.8
	*M. racemosus*	103.8 ± 1.2^bc^	100.8 ± 2.9^bc^	100.3 ± 1.9^b^	104.0 ± 1.0^c^	101.9 ± 1.0^bc^	104.5 ± 3.3^c^	92.8 ± 2.3^a^	101.4 ± 1.8^bc^	103.6 ± 3.9^bc^	99.0 ± 2.9^b^	105.2 ± 3.1^c^	101.6 ± 4.9
	*P. roqueforti*	16.2 ± 0.6^bc^	27.3 ± 5.4^cd^	14.9 ± 1.3^b^	20.2 ± 4.1^c^	9.5 ± 2.9^ab^	13.7 ± 2.9^b^	6.8 ± 0.5^a^	20.0 ± 2.3^c^	26.1 ± 1.4^cd^	33.1 ± 6.8^d^	33.8 ± 4.8^d^	20.2 ± 11.4
Growth rate	*C. inversicolor*	56.7 ± 14.0^ab^	50.9 ± 8.2^a^	79.8 ± 14.5^bcd^	78.1 ± 8.2^bcd^	59.7 ± 7.7^ab^	71.5 ± 9.2^bcd^	64.1 ± 6.0^abc^	80.6 ± 11.9^cd^	63.4 ± 6.3^abc^	75.5 ± 11.9^bcd^	78.0 ± 11.4^bcd^	69.3 ± 13.6
	*C. sinuosum*	98.0 ± 6.9^a^	104.2 ± 4.4^abc^	102.9 ± 3.7^abc^	106.8 ± 5.5^abc^	102.9 ± 3.7^abc^	111.7 ± 7.8^c^	99.5 ± 5.8^ab^	100.5 ± 6.3^abc^	101.3 ± 7.9^abc^	98.8 ± 4.4^a^	99.5 ± 5.8^ab^	102.4 ± 6.6
	*F. avenaceum*	109.5 ± 4.8^d^	96.1 ± 5.9^ab^	102.9 ± 5.9^abcd^	99.1 ± 4.1^abc^	104.1 ± 5.3^bcd^	100.5 ± 2.5^abc^	92.1 ± 5.9^a^	100.0 ± 1.0^abc^	109.6 ± 6.0^d^	100.6 ± 4.2^abcd^	103.7 ± 6.0^abcd^	101.9 ± 6.8
	*M. racemosus*	90.2 ± 4.6^bc^	83.1 ± 11.3^abc^	91.2 ± 4.5^c^	90.3 ± 2.2^bc^	82.2 ± 10.3^abc^	90.2 ± 3.5^bc^	75.8 ± 9.8^a^	87.5 ± 1.6^abc^	89.0 ± 3.9^bc^	86.8 ± 4.1^abc^	87.9 ± 4.5^abc^	86.8 ± 7.4
	*P. roqueforti*	85.7 ± 3.5^cd^	84.9 ± 3.7^bcd^	86.0 ± 4.1^cd^	86.0 ± 2.7^cd^	70.0 ± 6.1^a^	83.0 ± 6.2^bcd^	77.0 ± 6.3^ab^	82.0 ± 4.0^bcd^	85.0 ± 2.3^bcd^	85.5 ± 3.4^cd^	88.0 ± 3.1^d^	83.0 ± 6.3

*Values in the same row marked by different lowercase letters are significantly different using one-way ANOVA with Duncan’s test (≥95% confidence, *n* = 6). Clusters are based on chromosome polymorphism between the stains as determined by PFGE.*

For mycelium growth rates, corresponding to exponential growth phase of molds, cell-free supernatants of all *D. hansenii* strains induced inhibition of *C. inversicolor* (69.3%) while slight inhibition of *P. roqueforti* (83.0%) and *M. racemosus* (86.8%) was observed. Mycelium growth rates of *C. sinuosum* and *F. avenaceum* were not affected by any of the *D. hansenii* cell-free supernatants.

**FIGURE 2:**
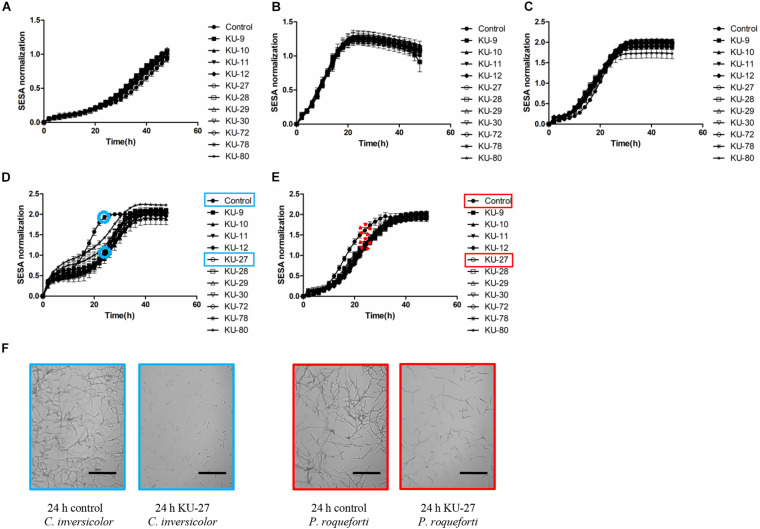
Effects of cell-free supernatants from *D. hansenii* strains KU-9, KU-10, KU-11, KU-12, KU-27, KU-28, KU-29, KU-30, KU-72, KU-78 and KU-80 on the growth of five mold species measured as Normalized SESA units using oCelloScope^TM^. **(A)** *Cladosporium sinuosum*, **(B)** *Fusarium avenaceum*, **(C)** *Mucor racemosus*, **(D)** *Cladosporium inversicolor*, **(E)** *Penicillium roqueforti*. Each spot represents the mean values from three independent experiments, and the error bars represent the standard deviation. **(F)** Examples of oCelloScope^TM^ images of growth measurements of *C. inversicolor* (blue) and *P. roqueforti* (red) in yeast peptone media (control) or in the presence of cell-free supernatants of *D. hansenii* strain KU-27 at 24 h. Scale bar: 300 μm.

The limited effect of cell-free supernatants collected from stationary phase cultures of *D. hansenii* strains were additionally confirmed by the growth curves of the molds (Figure 2). Hence, for *C. inversicolor* and *P. roqueforti*, the time point of reaching stationary phase was delayed by the *D. hansenii* cell-free supernatants, while no inhibitory effect was observed for *C. sinuosum*, *F. avenaceum*, and *M. racemosus*. Moreover, at the end of incubation (i.e., 48 h), the *D. hansenii* cell-free supernatants did not lead to reduced mycelium growth of any of the five tested molds. Overall, these results showed that the *D. hansenii* cell-free supernatants predominantly influenced the germination phase in a mold species dependent manner, rather than in a *D. hansenii* strain dependent manner.

### *Debaryomyces hansenii* Produced Antifungal VOCs in a Strain Dependent Manner

To elucidate the mechanisms of action involved in the antagonistic activities, the VOCs produced by stationary phase cultures of the 11 *D. hansenii* strains were determined. In total, 71 metabolites were detected by GC-MS and identified, i.e., 6 acids, 22 alcohols, 15 aldehydes, 3 benzene derivatives, 8 esters, 3 heterocyclic compounds, 12 ketones, and 2 phenols (Supplementary Table 2 lists all detected VOCs). The relative contents of each metabolite group are shown in Table 3. Variations in VOC contents were seen among the *D. hansenii* strains. Especially acids, alcohols, benzene derivatives, esters, ketones, and phenols were among the produced VOCs. Significantly decreased content of aldehydes was detected for all *D. hansenii* strains, except KU-80, while all *D. hansenii* strains had decreased content of heterocyclic compounds. Moreover, significant contents of acids and ketones were produced by *D. hansenii* strains (KU-12 and KU-30). While some *D. hansenii* strains produced significant contents of benzene derivatives (KU-9, KU-11, and KU-27), esters (KU-78, KU-9, KU-10, KU-29, and KU-27), and phenols (KU-11). Hence, strain-dependent VOCs profiles were obtained from the *D. hansenii* strains, however, no correlation between VOC profiles and the genetic clusters from the PFGE could be observed.

**TABLE 3:** Relative content of volatile organic compounds (VOCs) in each classification.

PFGE Cluster	Sample	Acids (*n* = 6)	Alcohols (*n* = 22)	Aldehydes (*n* = 15)	Benzene derivatives (*n* = 3)	Esters (*n* = 8)	Heterocyclic compounds (*n* = 3)	Ketones (*n* = 12)	Penols (*n* = 2)
	Control	0.06^a^	81.47^a^	14.56^c^	0.01^a^	1.90^ab^	0.15^e^	1.82^ab^	0.01^a^
I	KU-78	0.09^ab^	87.29^b^	6.65^b^	0.05^abc^	4.80^cd^	0.08^cd^	1.02^a^	0.02^ab^
I	KU-80	0.06^a^	81.28^a^	14.38^c^	0.05^abc^	2.67^ab^	0.05^a^	1.29^a^	0.01^a^
II	KU-12	0.14^b^	89.68^bc^	3.22^ab^	0.04^abc^	3.32^abc^	0.08^cd^	3.50^c^	0.01^a^
II	KU-30	0.13^b^	90.20^bc^	2.62^ab^	0.05^abc^	3.48^bc^	0.08^cd^	3.43^c^	0.02^ab^
II	KU-9	0.05^a^	90.50^bc^	2.47^ab^	0.06^bc^	5.36^d^	0.09^cd^	1.45^a^	0.02^ab^
II	KU-72	0.08^ab^	92.34^c^	3.09^ab^	0.03^abc^	1.69^a^	0.08^cd^	2.66^bc^	0.02^ab^
III	KU-11	0.11^ab^	91.94^c^	4.53^ab^	0.06^bc^	1.89^ab^	0.10^d^	1.32^a^	0.04^b^
III	KU-28	0.05^a^	93.19^c^	2.45^ab^	0.05^abc^	3.21^abc^	0.06^abc^	1.00^a^	0.01^a^
IV	KU-10	0.09^ab^	90.73^bc^	3.49^ab^	0.04^abc^	4.54^cd^	0.08^cd^	1.0^a^	0.01^a^
IV	KU-29	0.05^a^	92.15^c^	1.58^a^	0.03^abc^	5.30^d^	0.07^bc^	0.78^a^	0.03^ab^
IV	KU-27	0.08^ab^	88.97^bc^	3.77^ab^	0.07^c^	5.67^d^	0.05^ab^	1.40^a^	0.01^a^

*The relative percentage of relative peak area of each chemical class of samples, (n) refers to the number of volatile compounds in each class. Values in each classification marked by different lowercase letters are significantly different using one-way ANOVA with Duncan’s test (≥95% confidence, *n* = 2). Clusters are based on chromosome polymorphism between the stains as determined by PFGE.*

To better understand the correlation between the VOCs produced by the *D. hansenii* strains and their antifungal behaviors, Spearman’s rank correlation analysis was applied. The results of the correlation and coefficient analysis are shown in Table 4 and Supplementary Figure 3. Among the 71 VOCs, acetic acid, 3-methylbutanoic acid, and 2-pentanone exhibited a strong negative correlation with germination ratios for *C. inversicolor*. For *P. roqueforti*, 2-phenylethanol had a strong negative correlation with both germination ratio and mycelium growth rate, while acetone had a strong negative correlation with germination ratio. Thus, these five VOCs were chosen as targeted single VOCs in the following experiments.

**TABLE 4:** Spearman’s correlation coefficient analysis between germination ratio (%)/mycelium growth rate (μ/h) and the VOCs detected in the cell-free supernatants of *D. hansenii* strains.

Mold growth	Mold species	Volatile compounds	*R*-value (spearman)^*ab*^	Strength of association
Germination ratio	*C. inversicolor*	Acetic acid	−0.795	Strong
	*C. inversicolor*	3-methylbutanoic acid	−0.851	Strong
	*C. inversicolor*	2-pentanone	−0.731	Strong
	*P. roqueforti*	Acetone	−0.860	Strong
	*P. roqueforti*	2-phenylethanol	−0.753	Strong
Mycelium growth rate	*P. roqueforti*	2-phenylethanol	−0.881	Strong

*^*a*^*R* = 0 none; −0.3 ≤ *R* ≤ −0.1 week; −0.6 ≤ *R* ≤ −0.4 moderate; −0.9 ≤ *R* ≤ −0.7 strong; −1 perfect. ^*b*^The association was considered significant (*P* < 0.05), *P*-values of all compounds mentioned above were below 0.05.*

### Targeted Single VOCs Inhibited Germination, Mycelium Growth, and the Formation of Mycelial Pellicles of Contaminating Molds

**FIGURE 3:**
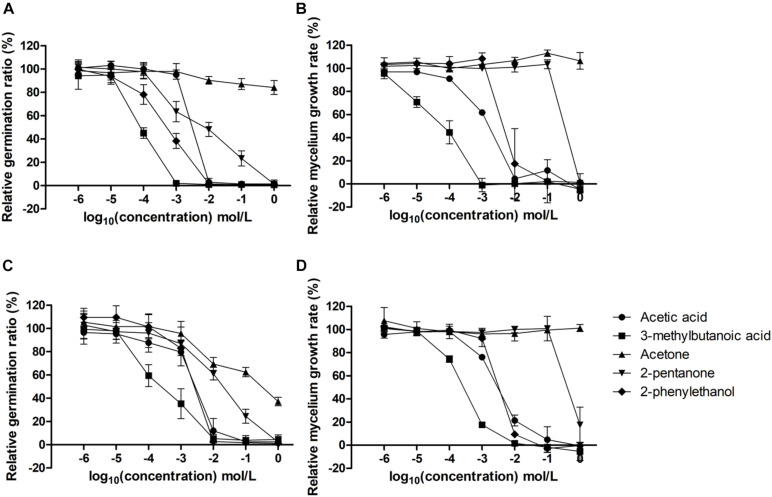
Inhibitory effects of targeted single VOCs on germination ratios (%) of *C. inversicolor* **(A)** and *P. roqueforti* **(C)** and mycelium growth rates (μ/h) of *C. inversicolor* **(B)** and *P. roqueforti* **(D)** at different concentrations (10^0^, 10^–1^, 10^–2^, 10^–3^, 10^–4^, 10^–5^, and 10^–6^ mol/L). Each spot represents the mean value from three independent experiments, and the error bars represent the standard deviations.

The inhibitory effects of targeted single VOCs were examined successively at different growth stages of *C. inversicolor* and *P. roqueforti*, i.e., on germination ratios and mycelium growth rates within 48 h (Figure 3 and Supplementary Figure 4). Based on germination ratios and mycelium growth rates in Figure 3, half-maximal inhibitory concentrations (IC_50_) were calculated for each growth stage, respectively (Table 5).

**TABLE 5:** IC_50_ of different VOCs for inhibiting germination and mycelium growth of *P. roqueforti* and *C. inversicolor*.

Mold growth	Volatile compounds	*C. inversicolor*	*P. roqueforti*
		IC_50_ (mol/L)	
Germination ratio	Acetic acid	2.47 × 10^–3^	2.80 × 10^–3^
	3-methylbutanoic acid	9.53 × 10^–5^	2.00 × 10^–4^
	Acetone	>1	9.21 × 10^–2^
	2-pentanone	1.06 × 10^–2^	2.60 × 10^–2^
	2-phenylethanol	1.99 × 10^–3^	1.73 × 10^–3^
Mycelium growth rate	Acetic acid	1.35 × 10^–3^	1.19 × 10^–3^
	3-methylbutanoic acid	6.32 × 10^–5^	2.67 × 10^–4^
	Acetone	>1	>1
	2-pentanone	9.45 × 10^–1^	9.51 × 10^–1^
	2-phenylethanol	7.49 × 10^–3^	3.45 × 10^–3^

For the germination ratios, gradually increasing concentration of 3-methylbutanoic acid, 2-phenylethanol, acetic acid, 2-pentanone, and acetone, respectively, were needed to obtain IC_50_ for *C. inversicolor* and *P. roqueforti*. For 3-methylbutanoic acid, IC_50_ were obtained at 9.53 × 10^–5^ and 2.00 × 10^–4^ mol/L for *C. inversicolor* and *P. roqueforti*, respectively. For 2-phenylethanol, IC_50_ were 1.99 × 10^–3^ and 1.73 × 10^–3^ mol/L for *C. inversicolor* and *P. roqueforti*, respectively. For acetic acid, IC_50_ were 2.47 × 10^–3^ and 2.80 × 10^–3^ mol/L for *C. inversicolor* and *P. roqueforti*, respectively. 2-pentanone had an inhibitory effect on germination ratios of *C. inversicolor* and *P. roqueforti* with an IC_50_ of 1.06 × 10^–2^ and 2.60 × 10^–2^ mol/L, respectively. Finally, acetone exhibited almost no inhibitory effect on germination ratio of *C. inversicolor* within the experimental period (48 h) but weakly inhibited germination ratio of *P. roqueforti* at IC_50_ of 9.21 × 10^–2^ mol/L.

For mycelium growth rates, gradually increasing concentrations of 3-methylbutanoic acid, acetic acid, 2-phenylethanol, 2-pentanone, and acetone, respectively, were needed to obtain IC_50_ for *C. inversicolor* and *P. roqueforti*. For 3-methylbutanoic acid, IC_50_ were 6.32 × 10^–5^ and 2.67 × 10^–4^ mol/L for *C. inversicolor* and *P. roqueforti*, respectively. For acetic acid, IC_50_ were 1.35 × 10^–3^ and 1.19 × 10^–3^ mol/L for *C. inversicolor* and *P. roqueforti*, respectively. For 2-phenylethanol, IC_50_ were 7.49 × 10^–3^ and 3.45 × 10^–3^ mol/L for *C. inversicolor* and *P. roqueforti*, respectively. Additionally, 2-pentanone showed a weak inhibitory effect on mycelium growth rate of *C. inversicolor* and *P. roqueforti*, whereas acetone exhibited no inhibitory effect on mycelium growth rates of the two tested molds.

Finally, for confirming the IC_50_ results as well as for evaluating long-term effects of exposure to different concentrations of the targeted VOCs, complete inhibition of mycelial pellicle formation was subsequently scored by visual inspections after 7 days incubation (Table 6). As expected, after long-term exposure to the targeted VOCs, inhibition of mycelial pellicle formation confirmed the IC_50_ results for both *C. inversicolor* and *P. roqueforti*. Hence, the highest inhibition was obtained for 3-methylbutanoic acid for both *C. inversicolor* and *P. roqueforti*, for which the concentrations were 10^–3^ and 10^–2^ mol/L, respectively. The concentrations of 2-phenylethanol and acetic acid for preventing the formation of mycelial pellicles were 10^–2^ and 10^–1^ mol/L for *C. inversicolor* and *P. roqueforti*, respectively. Furthermore, the concentration of 2-pentanone was 1 mol/L for *C. inversicolor*, while no inhibition on mycelial pellicle formation was observed for *P. roqueforti* after 7 days. Finally, acetone did not inhibit mycelial pellicle formation of either of the two molds after 7 days.

**TABLE 6:** Inhibitory effects of five VOCs on the formation of mycelial pellicles of molds after 7 days.

Mold species	Concentration (mol/L)	VOCs				
		Acetic acid	3-methylbutanoic acid	Acetone	2-pentanone	2-phenylethanol
***C. inversicolor***	10^0^	+++	+++	−	+++	+++
	10^–1^	+++	+++	−	−	+++
	10^–2^	++	+++	−	−	+
	10^–3^	−	++	−	−	−
	10^–4^	−	−	−	−	−
	10^–5^	−	−	−	−	−
	10^–6^	−	−	−	−	−
***P. roqueforti***	10^0^	+++	+++	−	−	+++
	10^–1^	+++	+++	−	−	+++
	10^–2^	−	+++	−	−	−
	10^–3^	−	−	−	−	−
	10^–4^	−	−	−	−	−
	10^–5^	−	−	−	−	−
	10^–6^	−	−	−	−	−

*+++ no mycelial pellicles formed. ++ less than or equal to 3 out of 6 replicates forming mycelial pellicles. + less than or equal to 5 out of 6 of replicates forming mycelial pellicles. − no inhibition.*

Among the targeted VOCs, estimated concentrations produced by the *D. hansenii* strains were calculated, when grown under the experimental conditions. The *D. hansenii* strains were found to produce estimated concentrations of 2-phenylethanol at 10^–3^ mol/L (data not shown), which was at the same concentration level as the detected IC_50_ for both *C. inversicolor* and *P. roqueforti.* The strains were additionally found to produce an estimated concentration of 10^–6^ mol/L for 3-methylbutanoic acid and 2-pentanone, and 10^–4^ mol/L for acetic acid and acetone (data not shown), which were all lower than the detected IC_50_ for both *C. invercolor* and *P. roqueforti* for the respective VOCs.

## Discussion

In this study, we evaluated *D. hansenii* strains isolated from Danish cheese brines for their antifungal activities against dairy contaminating molds. Our PFGE result confirmed the genetic heterogeneity of *D. hansenii* as previously reported (Petersen et al., 2002; Petersen and Jespersen, 2004). In Petersen et al. (2002), chromosome polymorphism among *D. hansenii* strains could be linked to different pH and NaCl tolerances. The variations in acidification or deacidification capabilities among the *D. hansenii* strains, found in the present study, could to some extend be linked to the genetic variance observed. The pH differences of the broth media (MYGP added with 4% (w/v) NaCl) might be related to *D. hansenii* producing or assimilating organic acids, proteolytic activity, production of ammonia, cell lysis, as well as several other factors (Gustafsson, 1979; Gancedo and Serrano, 1989; Freitas et al., 1999; Flores et al., 2004; Gori et al., 2007, 2012). Contrary, the VOCs profiles produced by the *D. hansenii* strains, in the present study, could not be linked to the specific chromosomal clusters as observed by PFGE, even though strain dependent differences in VOC production were observed. Strain-dependent VOC profiles have previously been observed among *D. hansenii* strains differing in M13 minisatellites, isolated from traditional ewe’s and goat’s cheese (Padilla et al., 2014b).

Among the five contaminating molds, *P. roqueforti* is, besides being a contaminant on surface ripened cheeses, a well known starter culture used in the production of blue-veined cheeses. *Mucor* spp. are well known on a variety of mold-ripened cheeses, especially from raw milk hard cheeses such as Saint Nectaire and Tomme de Savoie (Fox et al., 2004), and *M. racemosus* has especially been isolated from French cheeses (Hermet et al., 2015). Further, *F. avenaceum* has predominantly been reported as a mycotoxin producing pathogen on cereals, especially pronounced under cool and wet or humid conditions (Uhlig et al., 2007), however, it has also been isolated as a contaminating mold on Italian cheese (Montagna et al., 2004). Likewise, *F. avenaceum* was isolated at Danish dairies and identified to the species level in the present study. Finally, *Cladosporium* spp. have been isolated from traditional Italian cheese (De Santi et al., 2010). Originally isolated at Danish dairies, *C. inversicolor* and *C. sinuosum* were identified to species level, for the purpose of this study. However, *C. inversicolor* and *C. sinuosum* are so far mostly reported from plant materials (Bensch et al., 2015). Altogether, this indicates a huge species diversity among contaminating molds in dairy environments, far beyond what is usually investigated.

In the present study, the *D. hansenii* cell-free supernatants inhibited spore (conidia) germination and had only minor inhibitory effect on mold growth, indicating a fungistatic effect, which might be associated with the morphology of the spores or conidia of the five molds. Interestingly, the two *Cladosporium* spp., i.e., *C. inversicolor* and *C. sinuosum* responded very different to the *D. hansenii* cell-free supernatants. Germination and mycelium growth of *C. inversicolor* were significantly inhibited by cell-free supernatants of the *D. hansenii* strains, whereas we only observed a slight inhibition of germination for *C. sinuosum* and no effect on mycelium growth. The *Cladosporium* genus represents one of the larges genera of dematiaceous hyphomycetes and comprises several complexes, which are differentiated morphologically based on especially conidia morphology. *C. inversicolor* is in the *Cladosporium cladosporioides* complex and contains numerous conidia in long chains of up to eight conidia with walls typically being unthickened. *C. sinuosum* is in the *Cladosporium herbarum* complex and contains solitary conidia or short chains of up to four conidia which appear to be thick walled due to surface ornamentation (Bensch et al., 2015). These differences might explain some of the variance in the susceptibility observed between the two *Cladosporium* species.

Among the 71 VOCs produced by *D. hansenii*, five targeted VOCs (acetic acid, 3-methylbutanoic acid, acetone, 2-phenylethanol, and 2-pentanone) had a strong association with inhibition of germination and/or mycelium growth of *C. inversicolor* and *P. roqueforti* and were selected for detailed analyses. Accordingly, *D. hansenii* has been shown to produce acetic acid, acetone, 3-methylbutanoic acid, 2-pentanone, and 2-phenylethanol in cheese-like medium (Padilla et al., 2014a). Moreover, *D. hansenii* increased the amount of 2-pentanone in feta cheese (Bintsis and Robinson, 2004). In our study, 3-methylbutanoic acid had the strongest inhibitory effect and the lowest IC_50_ against germination and mycelium growth of *C. inversicolor* and *P. roqueforti* at 10^–5^ and 10^–4^mol/L, respectively. The sensory threshold for 3-methylbutanoic acid is reported to 120-700 ppb (1.17-6.85 × 10^–6^ mol/L) and at higher concentrations, this branched-chain fatty acid has been described to contribute with unpleasant odors as floor cloth, feet, and sweaty flavors in surface ripened cheeses from France (Lecanu et al., 2002). The estimated concentration of 3-methylbutanoic acid (10^–6^ mol/L) detected in the present study was therefore within the sensory thresholds but lower than the IC_50_ values mentioned above. Even so, the potential use of *D. hansenii* strains producing 3-methylbutanoic acid as a biocontrol agent is still interesting. Higher levels of 3-methylbutanoic acid might potentially be obtained under different processing conditions, as reported by Durá et al. (2004) where NaCl and lactate increased the levels of 3-methylbutanoic acid produced by *D. hansenii*, and detailed analyses still need to be carried out in cheese under relevant maturation conditions.

The VOC 2-phenylethanol is reported as a common inhibitory compound produced by several biocontrol yeasts including *Debaryomyces nepalensis*, *Wickerhamomyces anomalus*, *Metschnikowia pulcherrima*, *Saccharomyces cerevisiae*, and *Pichia manshurica* (formerly, *Pichia galeiformis*) (Zhou et al., 2018; Contarino et al., 2019; Chen et al., 2020). In this study, we found a strong inhibitory effect of 2-phenylethanol on germination and mycelium growth of *C. inversicolor* and *P. roqueforti* at 10^–3^ mol/L. Accordingly, we have previously found that 2-phenylethanol completely impaired conidia germination, hyphal membrane integrity, and growth of another two food spoilage molds, i.e., *Penicillium expansum* and *Penicillium nordicum* at 15 × 10^–3^ mol/L (Huang et al., 2020). The concentration of 2-phenylethanol produced by *D. hansenii* strains in the present study was approximately 10^–3^ mol/L and therefore comparable with the IC_50_ (10^–3^ mol/L level), thus highlighting the potential application of the *D. hansenii* strains for biological production of 2-phenylethanol as an antifungal agent. Moreover, 2-phenylethanol is a well-known quorum sensing molecule produced by *D. hansenii*, and is found to upregulate traits involved in adhesion and sliding motility of *D. hansenii* (Gori et al., 2011), increasing the colonization probability on cheese surfaces (Mortensen et al., 2005; Gori et al., 2011), which in turn could contribute to the quality of the final product.

Acetic acid is a common antimicrobial compound and its antifungal activity is well investigated (Cabo et al., 2002; Fernandez et al., 2017; Guimarães et al., 2018; Sadiq et al., 2019). In this study, the IC_50_ for acetic acid was found at 10^–3^ mol/L against *C. inversicolor* and *P. roqueforti*. Quattrini et al. (2019) reported the minimum inhibitory concentration (MIC) of acetic acid for *P. roqueforti* growth to 2.5 ± 0.6 × 10^–2^ mol/L after 5 days, which is somewhat lower than the concentration of acetic acid for preventing mycelial pellicle formation of *P. roqueforti* (approximately 10^–1^ mol/L) after 7 days, in the present study. In Quattrini et al. (2019), MIC was determined after 5 days in conditions mimicking sourdough using modified MRS, while in the present study inhibition was scored after 7 days in MEB, a medium for optimal mold growth. These differences could explain the different concentrations determined.

In soft cheeses, 2-pentanone has been detected in Camembert, Vacherin, Limburger, Trappist, Gorgonzola and blue cheese above the odor threshold of 0.5-61 ppm contributing fruity, acetone, sweet, and ethereal odor (Sablé and Cottenceau, 1999). In the current study, 2-pentanone showed a weak inhibitory effect on the germination and mycelium growth of *C. inversicolor* and *P. roqueforti*. Further, the estimated concentration of 2-pentanone produced by *D. hansenii* in this study, was lower than the detected IC_50_ but within the ranges of odor threshold reported in previous studies (Sablé and Cottenceau, 1999).

The results of the present study indicate that the antifungal activity of *D. hansenii* is complex and likely results from a synergistic and or additive effect of several inhibitory VOCs, rather than the effect of single compounds. Similarly, growth inhibition of postharvest pathogenic molds in packaged fresh fruit by the proven biocontrol yeasts *W. anomalus* BS91 and *M. pulcherrima* MPR3 has been suggested to arise from a synergistic effect of VOCs and CO_2_ (Contarino et al., 2019). Moreover, inhibition of *Fusarium* spp. growth in the presence of cell-free supernatants of *Lactiplantibacillus plantarum* (formerly, *Lactobacillus plantarum*) has been shown not to arise alone from the lactic acid being produced by the lactic acid bacteria (Laitila et al., 2002). Further, additional mechanisms, not investigated in the current study, might also add to the combined antifungal activity of *D. hansenii*, including non-volatile compounds like killer toxins (Banjara et al., 2016), and competition for nutrients and space (Spadaro and Droby, 2016). As nutrients and space are generally limited in cheese processing (Irlinger and Mounier, 2009), this could be especially relevant. Interestingly, *D. hansenii* isolated from dry-cured meat products has been reported to inhibit toxigenic penicillia in co-culture assays on solid media, which could not be fully reproduced by cell-free supernatants or mouth-to-mouth assays. This indicates that efficient mold inhibition relied on additive or synergistic effects of the yeast inhibition factors such as competition for nutrients and space as well as production of soluble compounds or VOCs (Núñez et al., 2015). Similarly, *P. manshurica* has been shown to colonize and amplify quickly in citrus wounds and further to produce eight VOCs, including acetic acid and 2-phenylethanol during growth, which inhibited growth of *Penicillium digitatum*, causing citrus green mold (Chen et al., 2020).

In conclusion, genetic differences of *D. hansenii* together with diverse acidifying or deacidifying capability were confirmed. Cell-free supernatants of *D. hansenii* strains exhibited a strong inhibitory effect on *C. inversicolor* and *P. roqueforti*. Using DHS-GC-MS, 71 VOCs produced by *D. hansenii* strains were identified. Among the VOCs, strong correlation between inhibition of germination of *C. inversicolor* and *P. roqueforti* were obtained for five VOCs (acetic acid, acetone, 3-methylbutanoic acid, 2-pentanone, and 2-phenylethanol), while 2-phenylethanol also correlated strongly with inhibition of mycelium growth of *P. roqueforti*. 3-methylbutanoic acid showed the strongest inhibitory effects on germination and mycelium growth of the two mold species, i.e., IC_50_ of 10^–5^ and 10^–4^ mol/L, respectively. 2-phenylethanol and acetic acid also exhibited strong inhibitory effects on germination and mycelium growth at IC_50_ of 10^–3^ mol/L. Relative weak inhibitory effects of 2-pentanone and acetone were obtained in this study. The results from the current study point to an additive and possibly synergistic effect of the antifungal compounds produced by *D. hansenii*, which needs to be further studied. From an overall perspective, it can be concluded that some native *D. hansenii* strains in the dairy manufacturing environment have a so far neglected role in the natural preservation of cheeses and are eligible for biocontrol of contaminating molds in cheese production. The results additionally point to the coming use of halophilic *D. hansenii* strains as biocontrol agents to be added as bioprotective cultures in the dairy industry, e.g., as an additive to cheese brines.

## Data Availability Statement

The original contributions presented in the study are included in the article/Supplementary Material, further inquiries can be directed to the corresponding author/s.

## Author Contributions

CH, LZ, PJ, NA, and LJ conceived and designed the study. MP contributed to the GC-MS analyses. CH performed the experiments and drafted the manuscript. PJ, NA, and LJ revised the manuscript. All authors contributed to the article and approved the submitted version.

## Conflict of Interest

The authors declare that the research was conducted in the absence of any commercial or financial relationships that could be construed as a potential conflict of interest.

## Footnotes

1 http://www.combase.cc/index.php/en/

## References

[B1] Akoglu H. (2018). User’s guide to correlation coefficients. *Turkish J. Emerg. Med.* 18 91–93. 10.1016/j.tjem.2018.08.001 30191186 PMC6107969

[B2] Ando H. Hatanaka K. Ohata I. Yamashita-Kitaguchi Y. Kurata A. Kishimoto N. (2012). Antifungal activities of volatile substances generated by yeast isolated from Iranian commercial cheese. *Food Control* 26 472–478. 10.1016/j.foodcont.2012.02.017

[B3] Andrade M. J. Thorsen L. Rodríguez A. Córdoba J. J. Jespersen L. (2014). Inhibition of ochratoxigenic moulds by *Debaryomyces hansenii* strains for biopreservation of dry-cured meat products. *Int. J. Food Microbiol.* 170 70–77. 10.1016/j.ijfoodmicro.2013.11.004 24291184

[B4] Banjara N. Nickerson K. W. Suhr M. J. Hallen-Adams H. E. (2016). Killer toxin from several food-derived *Debaryomyces hansenii* strains effective against pathogenic Candida yeasts. *Int. J. Food Microbiol.* 222 23–29. 10.1016/j.ijfoodmicro.2016.01.016 26828815

[B5] Baranyi J. Roberts T. A. (1994). A dynamic approach to predicting bacterial growth in food. *Int. J. Food Microbiol.* 23 277–294. 10.1016/0168-1605(94)90157-0 7873331

[B6] Bencheqroun S. K. Bajji M. Massart S. Labhilili M. Jaafari S. E. Jijakli M. H. (2007). *In vitro* and *in situ* study of postharvest apple blue mold biocontrol by *Aureobasidium pullulans*: evidence for the involvement of competition for nutrients. *Postharvest Biol. Technol.* 46 128–135. 10.1016/j.postharvbio.2007.05.005 17390872

[B7] Bensch K. Groenewald J. Z. Braun U. Dijksterhuis J. de Jesús Yáñez-Morales M. Crous P. W. (2015). Common but different: the expanding realm of *Cladosporium*. *Stud. Mycol.* 82 23–74. 10.1016/j.simyco.2015.10.001 26955200 PMC4774271

[B8] Bintsis T. Robinson R. K. (2004). A study of the effects of adjunct cultures on the aroma compounds of Feta-type cheese. *Food Chem.* 88 435–441. 10.1016/j.foodchem.2004.01.057

[B9] Cabo M. L. Braber A. F. Koenraad P. M. F. J. (2002). Apparent antifungal activity of several lactic acid bacteria against *Penicillium discolor* is due to acetic acid in the medium. *J. Food Prot.* 65 1309–1316. 10.4315/0362-028X-65.8.1309 12182485

[B10] Chen O. Yi L. Deng L. Ruan C. Zeng K. (2020). Screening antagonistic yeasts against citrus green mold and the possible biocontrol mechanisms of *Pichia galeiformis* (BAF03). *J. Sci. Food Agric.* 100 3812–3821. 10.1002/jsfa.10407 32248529

[B11] Choińska R. Piasecka-Jóźwiak K. Chabłowska B. Dumka J. Łukaszewicz A. (2020). Biocontrol ability and volatile organic compounds production as a putative mode of action of yeast strains isolated from organic grapes and rye grains. *Antonie van Leeuwenhoek Int. J. Gen. Mol. Microbiol.* 113 1135–1146. 10.1007/s10482-020-01420-7 32372375 PMC7334268

[B12] Contarino R. Brighina S. Fallico B. Cirvilleri G. Parafati L. Restuccia C. (2019). Volatile organic compounds (VOCs) produced by biocontrol yeasts. *Food Microbiol.* 82 70–74. 10.1016/j.fm.2019.01.008 31027821

[B13] Czarnecka M. Żarowska B. Połomska X. Restuccia C. Cirvilleri G. (2019). Role of biocontrol yeasts *Debaryomyces hansenii* and Wickerhamomyces anomalus in plants’ defence mechanisms against Monilinia fructicola in apple fruits. *Food Microbiol.* 83 1–8. 10.1016/j.fm.2019.04.004 31202399

[B14] De Santi M. Sisti M. Barbieri E. Piccoli G. Brandi G. Stocchi V. (2010). A combined morphologic and molecular approach for characterizing fungal microflora from a traditional Italian cheese (Fossa cheese). *Int. Dairy J.* 20 465–471. 10.1016/j.idairyj.2010.02.004

[B15] Durá M. A. Flores M. Toldrá F. (2004). Effect of growth phase and dry-cured sausage processing conditions on *Debaryomyces* spp. generation of volatile compounds from branched-chain amino acids. *Food Chem.* 86 391–399. 10.1016/j.foodchem.2003.09.014

[B16] Fernandez B. Vimont A. Desfossés-Foucault É Daga M. Arora G. Fliss I. (2017). Antifungal activity of lactic and propionic acid bacteria and their potential as protective culture in cottage cheese. *Food Control* 78 350–356. 10.1016/j.foodcont.2017.03.007

[B17] Fialho M. B. Toffano L. Pedroso M. P. Augusto F. Pascholati S. F. (2010). Volatile organic compounds produced by Saccharomyces cerevisiae inhibit the in vitro development of *Guignardia citricarpa*, the causal agent of citrus black spot. *World J. Microbiol. Biotechnol.* 26 925–932. 10.1007/s11274-009-0255-4

[B18] Flores M. Durá M. A. Marco A. Toldrá F. (2004). Effect of *Debaryomyces* spp. on aroma formation and sensory quality of dry-fermented sausages. *Meat Sci.* 68 439–446. 10.1016/j.meatsci.2003.04.001 22062412

[B19] Fox P. F. McSweeney P. L. Cogan T. M. Guinee T. P. (2004). “General aspects,” in *Cheese: Chemistry, Physics and Microbiology*, Vol. 1 eds Fox P. F. McSweeney P. L. Cogan T. M. Guinee T. P. (London: Elsevier), 1–18.

[B20] Fredborg M. Andersen K. R. Jørgensen E. Droce A. Olesen T. Jensen B. B. (2013). Real-time optical antimicrobial susceptibility testing. *J. Clin. Microbiol.* 51 2047–2053. 10.1128/JCM.00440-13 23596243 PMC3697729

[B21] Freimoser F. M. Rueda-Mejia M. P. Tilocca B. Migheli Q. (2019). Biocontrol yeasts: mechanisms and applications. *World J. Microbiol. Biotechnol.* 35 1–19. 10.1007/s11274-019-2728-4 31576429 PMC6773674

[B22] Freitas A. C. Pintado A. E. Pintado M. E. Malcata F. X. (1999). Organic acids produced by lactobacilli, enterococci and yeasts isolated from Picante cheese. *Eur. Food Res. Technol.* 209 434–438. 10.1007/s002170050522

[B23] Fröhlich-Wyder M. T. Arias-Roth E. Jakob E. (2019). Cheese yeasts. *Yeast* 36 129–141. 10.1002/yea.3368 30512214

[B24] Gancedo C. Serrano R. (1989). “Energy-yielding metabolism,” in *The Yeasts*, eds Rose J. S. Harrison A. H. (London&New York, NY: Academic Press), 205–259.

[B25] Garnier L. Valence F. Mounier J. (2017). Diversity and control of spoilage fungi in dairy products: an update. *Microorganisms* 5:42. 10.3390/microorganisms5030042 28788096 PMC5620633

[B26] Gori K. Knudsen P. B. Nielsen K. F. Arneborg N. Jespersen L. (2011). Alcohol-based quorum sensing plays a role in adhesion and sliding motility of the yeast *Debaryomyces hansenii*. *FEMS Yeast Res.* 11 643–652. 10.1111/j.1567-1364.2011.00755.x 22093748

[B27] Gori K. Mortensen H. D. Arneborg N. Jespersen L. (2007). Ammonia production and its possible role as a mediator of communication for *Debaryomyces hansenii* and other cheese-relevant yeast species. *J. Dairy Sci.* 90 5032–5041. 10.3168/jds.2006-750 17954742

[B28] Gori K. Sørensen L. M. Petersen M. A. Jespersen L. Arneborg N. (2012). *Debaryomyces hansenii* strains differ in their production of flavor compounds in a cheese-surface model. *Microbiologyopen* 1 161–168. 10.1002/mbo3.11 22950022 PMC3426413

[B29] Grzegorczyk M. Żarowska B. Restuccia C. Cirvilleri G. (2017). Postharvest biocontrol ability of killer yeasts against *Monilinia fructigena* and Monilinia fructicola on stone fruit. *Food Microbiol.* 61 93–101. 10.1016/j.fm.2016.09.005 27697174

[B30] Guimarães A. Venancio A. Abrunhosa L. (2018). Antifungal effect of organic acids from lactic acid bacteria on *Penicillium nordicum*. *Food Addit. Contam. Part A Chem. Anal. Control. Expo. Risk Assess.* 35 1803–1818. 10.1080/19440049.2018.1500718 30016195

[B31] Gustafsson L. (1979). The ATP pool in relation to the production of glycerol and heat during growth of the halotolerant yeast *Debaryomyces hansenii*. *Arch. Microbiol.* 120 15–23. 10.1007/BF00413266

[B32] Haastrup M. K. Johansen P. Malskær A. H. Castro-Mejía J. L. Kot W. Krych L. (2018). Cheese brines from Danish dairies reveal a complex microbiota comprising several halotolerant bacteria and yeasts. *Int. J. Food Microbiol.* 285 173–187. 10.1016/j.ijfoodmicro.2018.08.015 30176565

[B33] Hermet A. Méheust D. Mounier J. Barbier G. Jany J. L. (2015). Molecular systematics in the genus Mucor with special regards to species encountered in cheese. *Fungal Biol.* 119:857. 10.1016/j.funbio.2015.06.001 22658314

[B34] Hernandez-Montiel L. G. Gutierrez-Perez E. D. Murillo-Amador B. Vero S. Chiquito-Contreras R. G. Rincon-Enriquez G. (2018). Mechanisms employed by *Debaryomyces hansenii* in biological control of anthracnose disease on papaya fruit. *Postharvest Biol. Technol.* 139 31–37. 10.1016/j.postharvbio.2018.01.015

[B35] Hernández-Montiel L. G. Ochoa J. L. Troyo-Diéguez E. Larralde-Corona C. P. (2010). Biocontrol of postharvest blue mold (*Penicillium italicum Wehmer*) on Mexican lime by marine and citrus *Debaryomyces hansenii* isolates. *Postharvest Biol. Technol.* 56 181–187. 10.1016/j.postharvbio.2009.12.010

[B36] Huang C. Qian Y. Viana T. Siegumfeldt H. Arneborg N. Larsen N. (2020). The quorum-sensing molecule 2-phenylethanol impaired conidial germination, hyphal membrane integrity and growth of *Penicillium expansum* and *Penicillium nordicum*. *J. Appl. Microbiol.* 129 278–286. 10.1111/jam.14621 32097516

[B37] Irlinger F. Mounier J. (2009). Microbial interactions in cheese: implications for cheese quality and safety. *Curr. Opin. Biotechnol.* 20 142–148. 10.1016/j.copbio.2009.02.016 19342218

[B38] Koutsoumanis K. Allende A. Alvarez-Ordóñez A. Bolton D. Bover-Cid S. Chemaly M. (2020). Update of the list of QPS-recommended biological agents intentionally added to food or feed as notified to EFSA 12: suitability of taxonomic units notified to EFSA until March 2020. *EFSA J.* 18:e06174. 10.2903/j.efsa.2020.6174 32760463 PMC7331632

[B39] Kure C. F. Skaar I. (2019). The fungal problem in cheese industry. *Curr. Opin. Food Sci.* 29 14–19. 10.1016/j.cofs.2019.07.003

[B40] Laitila A. Alakomi H. L. Raaska L. Mattila-Sandholm T. Haikara A. (2002). Antifungal activities of two Lactobacillus plantarum strains against *Fusarium* moulds in vitro and in malting of barley. *J. Appl. Microbiol.* 93 566–576. 10.1046/j.1365-2672.2002.01731.x 12234339

[B41] Lecanu L. Ducruet V. Jouquand C. Gratadoux J. J. Feigenbaum A. (2002). Optimization of headspace solid-phase microextraction (SPME) for the odor analysis of surface-ripened cheese. *J. Agric. Food Chem.* 50 3810–3817. 10.1021/jf0117107 12059164

[B42] Lessard M. H. Bélanger G. St-Gelais D. Labrie S. (2012). The composition of camembert cheese-ripening cultures modulates both mycelial growth and appearance. *Appl. Environ. Microbiol.* 78 1813–1819. 10.1128/AEM.06645-11 22247164 PMC3298135

[B43] Liu J. Sui Y. Wisniewski M. Droby S. Liu Y. (2013). Review: utilization of antagonistic yeasts to manage postharvest fungal diseases of fruit. *Int. J. Food Microbiol.* 167 153–160. 10.1016/j.ijfoodmicro.2013.09.004 24135671

[B44] Liu J. Toldam-Andersen T. B. Petersen M. A. Zhang S. Arneborg N. Bredie W. L. P. (2015). Instrumental and sensory characterisation of Solaris white wines in Denmark. *Food Chem.* 166 133–142. 10.1016/j.foodchem.2014.05.148 25053038

[B45] Liu S. Q. Tsao M. (2009). Biocontrol of dairy moulds by antagonistic dairy yeast *Debaryomyces hansenii* in yoghurt and cheese at elevated temperatures. *Food Control* 20 852–855. 10.1016/j.foodcont.2008.10.006

[B46] Liu S. Q. Tsao M. (2010). Biocontrol of spoilage yeasts and moulds by Williopsis saturnus var. saturnus in yoghurt. *Nutr. Food Sci.* 40 166–175. 10.1108/00346651011029192

[B47] Masoud W. Poll L. Jakobsen M. (2005). Influence of volatile compounds produced by yeasts predominant during processing of Coffea arabica in East Africa on growth and ochratoxin A (OTA) production by *Aspergillus ochraceus*. *Yeast* 22 1133–1142. 10.1002/yea.1304 16240461

[B48] Medina-Córdova N. López-Aguilar R. Ascencio F. Castellanos T. Campa-Córdova A. I. Angulo C. (2016). Biocontrol activity of the marine yeast *Debaryomyces hansenii* against phytopathogenic fungi and its ability to inhibit mycotoxins production in maize grain (*Zea mays* L.). *Biol. Control* 97 70–79. 10.1016/j.biocontrol.2016.03.006

[B49] Medina-Córdova N. Rosales-Mendoza S. Hernández-Montiel L. G. Angulo C. (2018). The potential use of *Debaryomyces hansenii* for the biological control of pathogenic fungi in food. *Biol. Control* 121 216–222. 10.1016/j.biocontrol.2018.03.002

[B50] Montagna M. T. Santacroce M. P. Spilotros G. Napoli C. Minervini F. Papa A. (2004). Investigation of fungal contamination in sheep and goat cheeses in southern Italy. *Mycopathologia* 158 245–249. 10.1023/B:MYCO.0000041897.17673.2c 15518354

[B51] Mortensen H. D. Gori K. Jespersen L. Arneborg N. (2005). *Debaryomyces hansenii* strains with different cell sizes and surface physicochemical properties adhere differently to a solid agarose surface. *FEMS Microbiol. Lett.* 249 165–170. 10.1016/j.femsle.2005.06.009 16002242

[B52] Núñez F. Lara M. S. Peromingo B. Delgado J. Sánchez-Montero L. Andrade M. J. (2015). Selection and evaluation of *Debaryomyces hansenii* isolates as potential bioprotective agents against toxigenic penicillia in dry-fermented sausages. *Food Microbiol.* 46 114–120. 10.1016/j.fm.2014.07.019 25475274

[B53] Padilla B. Belloch C. López-Díez J. J. Flores M. Manzanares P. (2014a). Potential impact of dairy yeasts on the typical flavour of traditional ewes’ and goats’ cheeses. *Int. Dairy J.* 35 122–129. 10.1016/j.idairyj.2013.11.002

[B54] Padilla B. Manzanares P. Belloch C. (2014b). Yeast species and genetic heterogeneity within *Debaryomyces hansenii* along the ripening process of traditional ewes’ and goats’ cheeses. *Food Microbiol.* 38 160–166. 10.1016/j.fm.2013.09.002 24290639

[B55] Petersen K. M. Jespersen L. (2004). Genetic diversity of the species *Debaryomyces hansenii* and the use of chromosome polymorphism for typing of strains isolated from surface-ripened cheeses. *J. Appl. Microbiol.* 97 205–213. 10.1111/j.1365-2672.2004.02293.x 15186457

[B56] Petersen K. M. Westall S. Jespersen L. (2002). Microbial succession of *Debaryomyces hansenii* strains during the production of Danish surfaced-ripened cheeses. *J. Dairy Sci.* 85 478–486. 10.3168/jds.S0022-0302(02)74098-8 11949849

[B57] Quattrini M. Liang N. Fortina M. G. Xiang S. Curtis J. M. Gänzle M. (2019). Exploiting synergies of sourdough and antifungal organic acids to delay fungal spoilage of bread. *Int. J. Food Microbiol.* 302 8–14. 10.1016/j.ijfoodmicro.2018.09.007 30220438

[B58] Sablé S. Cottenceau G. (1999). Current knowledge of soft cheeses flavor and related compounds. *J. Agric. Food Chem.* 47 4825–4836. 10.1021/jf990414f 10606538

[B59] Sadiq F. A. Yan B. Tian F. Zhao J. Zhang H. Chen W. (2019). Lactic acid bacteria as antifungal and anti-mycotoxigenic agents: a comprehensive review. *Compr. Rev. Food Sci. Food Saf.* 18 1403–1436. 10.1111/1541-4337.12481 33336904

[B60] Santos A. Marquina D. (2004). Killer toxin of Pichia membranifaciens and its possible use as a biocontrol agent against grey mould disease of grapevine. *Microbiology* 150 2527–2534. 10.1099/mic.0.27071-0 15289549

[B61] Sørensen L. M. Gori K. Petersen M. A. Jespersen L. Arneborg N. (2011). Flavour compound production by Yarrowia lipolytica, Saccharomyces cerevisiae and *Debaryomyces hansenii* in a cheese-surface model. *Int. Dairy J.* 21 970–978. 10.1016/j.idairyj.2011.06.005

[B62] Spadaro D. Droby S. (2016). Development of biocontrol products for postharvest diseases of fruit: The importance of elucidating the mechanisms of action of yeast antagonists. *Trends Food Sci. Technol.* 47 39–49. 10.1016/j.tifs.2015.11.003

[B63] Trinci A. P. J. (1971). Exponential growth of the germ tubes of fungal spores. *J. Gen. Microbiol.* 67 345–348. 10.1099/00221287-67-3-345

[B64] Uhlig S. Jestoi M. Parikka P. (2007). *Fusarium avenaceum* - The North European situation. *Int. J. Food Microbiol.* 119 17–24. 10.1016/j.ijfoodmicro.2007.07.021 17884217

[B65] Van Den Tempel T. Jakobsen M. (2000). The technological characteristics of *Debaryomyces hansenii* and *Yarrowia lipolytica* and their potential as starter cultures for production of Danablu. *Int. Dairy J.* 10 263–270. 10.1016/S0958-6946(00)00053-4

[B66] Zhang L. Huang C. Malskær A. H. Jespersen L. Arneborg N. Johansen P. G. (2020). The effects of NaCl and temperature on growth and survival of yeast strains isolated from Danish cheese brines. *Curr. Microbiol.* 77 3377–3384. 10.1007/s00284-020-02185-y 32936341

[B67] Zhou Y. Li W. Zeng J. Shao Y. (2018). Mechanisms of action of the yeast *Debaryomyces nepalensis* for control of the pathogen Colletotrichum gloeosporioides in mango fruit. *Biol. Control* 123 111–119. 10.1016/j.biocontrol.2018.05.014

