# Supplementary material for: Debaryomyces hansenii Strains Isolated From Danish Cheese Brines Act as Biocontrol Agents to Inhibit Germination and Growth of Contaminating Molds
Source: Front Microbiol. 2021 Jun 15;12:662785. doi: 10.3389/fmicb.2021.662785 (PMC8239395; doi:10.3389/fmicb.2021.662785)
Supplement: Supplementary file 1 [file Image_1.PDF]

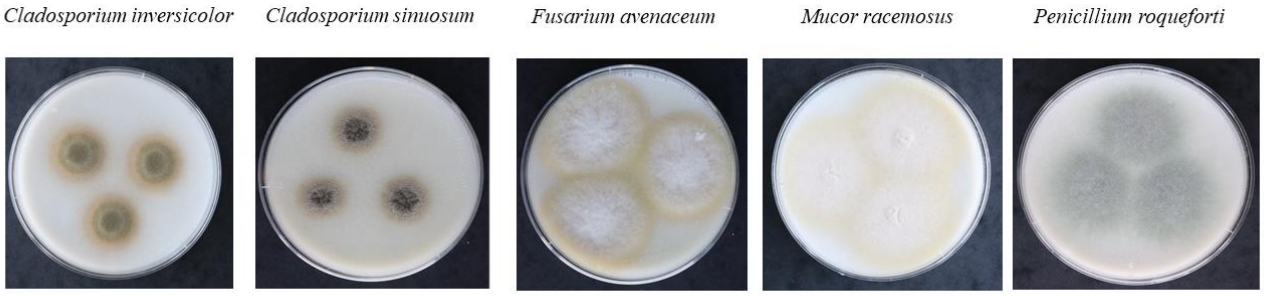

Supplementary Figure 1 Pictures of the molds grown on cheese agar after 7 days at 25 °C.
