# Supplementary material for: Debaryomyces hansenii Strains Isolated From Danish Cheese Brines Act as Biocontrol Agents to Inhibit Germination and Growth of Contaminating Molds
Source: Front Microbiol. 2021 Jun 15;12:662785. doi: 10.3389/fmicb.2021.662785 (PMC8239395; doi:10.3389/fmicb.2021.662785)
Supplement: Supplementary file 2 [file Image_4.PDF]

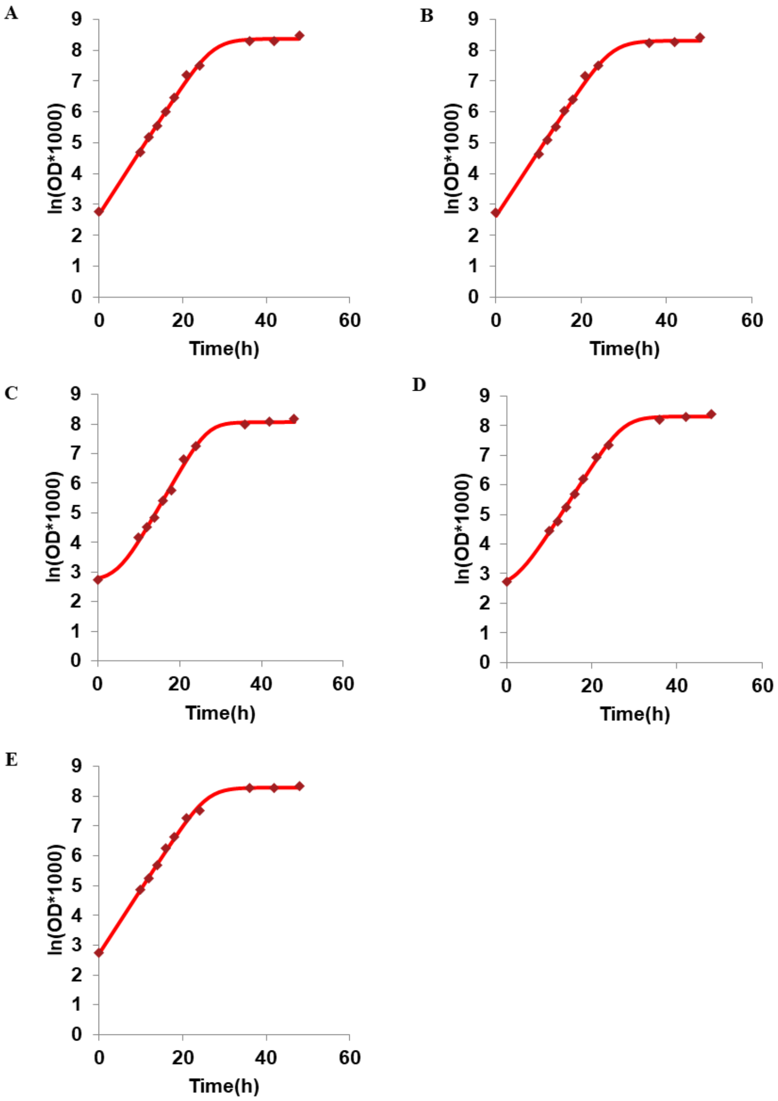

Supplementary Figure 2 Growth curves of five *D. hansenii* strains measured by spectrophotometer and analysed by DMfit software. **(A)** KU-9, **(B)** KU-11, **(C)** KU-12, **(D)** KU-27, and **(E)** KU-28. Measurements are means of duplicates.
