# Supplementary material for: Debaryomyces hansenii Strains Isolated From Danish Cheese Brines Act as Biocontrol Agents to Inhibit Germination and Growth of Contaminating Molds
Source: Front Microbiol. 2021 Jun 15;12:662785. doi: 10.3389/fmicb.2021.662785 (PMC8239395; doi:10.3389/fmicb.2021.662785)
Supplement: Supplementary file 3 [file Image_2.PDF]

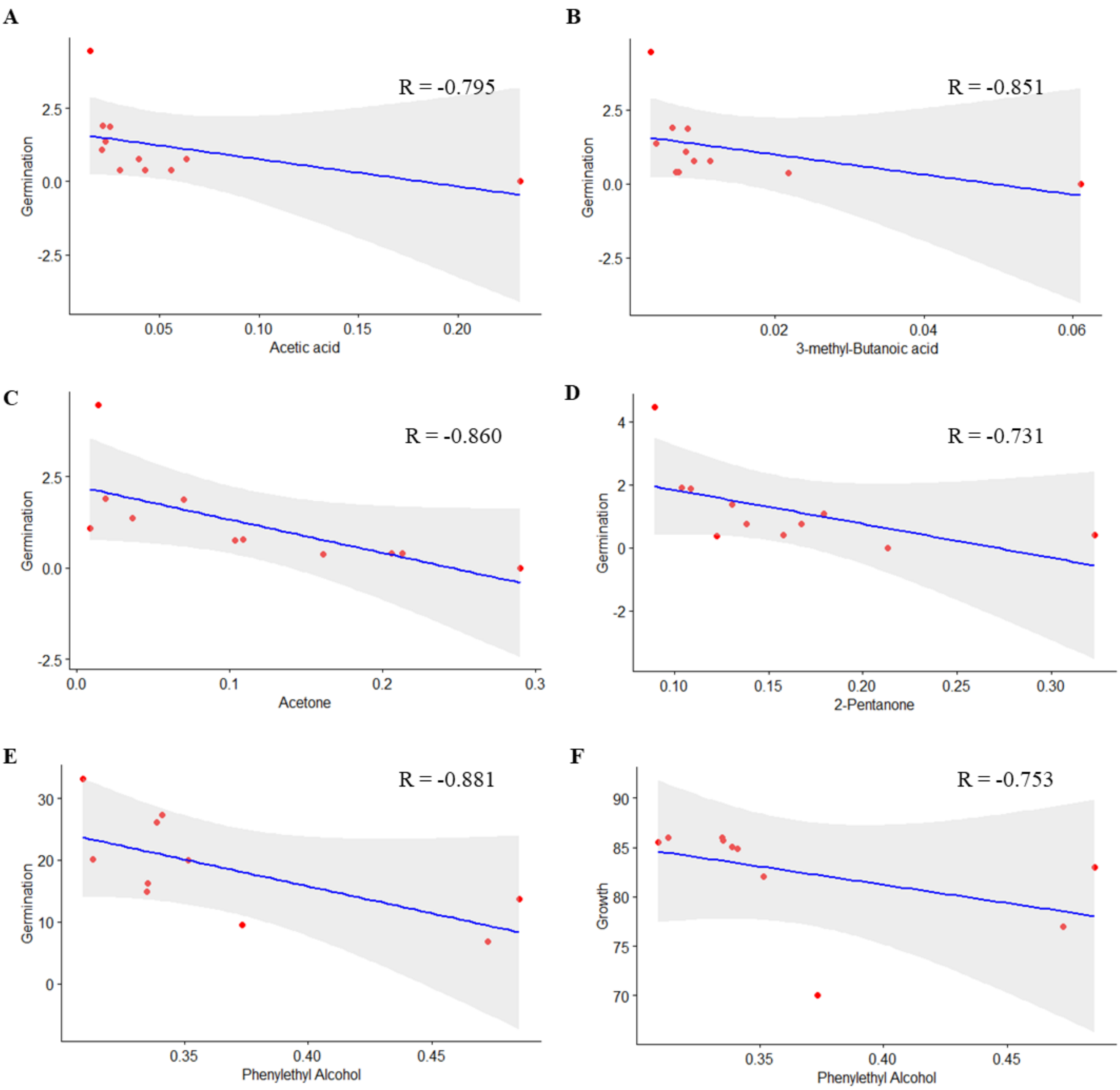

Supplementary Figure 3 Scatterplots of spearman's correlation coefficients between relative germination ratios or mycelium growth rate and relative peak area of the compounds. **(A)** Y-axis: germination rate of *P. roqueforti*; X-axis: peak area of acetic acid; **(B)** Y-axis: germination rate of *P. roqueforti*; X-axis: peak area of 3-methyl-butanoic acid; **(C)** Y-axis: germination rate of *C. inversicolor*; X-axis: peak area of acetone, **(D)** Y-axis: germination rate of *C. inversicolor*; X-axis: peak area of 2-pentanoe; **(E)** Y-axis: germination rate of *C. inversicolor*; X-axis: peak area of 2-phenylethanol; **(F)** Y-axis: mycelium growth rate of *C. inversicolor*; X-axis: peak area of 2-phenylethanol.
