# Supplementary material for: Debaryomyces hansenii Strains Isolated From Danish Cheese Brines Act as Biocontrol Agents to Inhibit Germination and Growth of Contaminating Molds
Source: Front Microbiol. 2021 Jun 15;12:662785. doi: 10.3389/fmicb.2021.662785 (PMC8239395; doi:10.3389/fmicb.2021.662785)
Supplement: Supplementary file 4 [file Image_3.PDF]

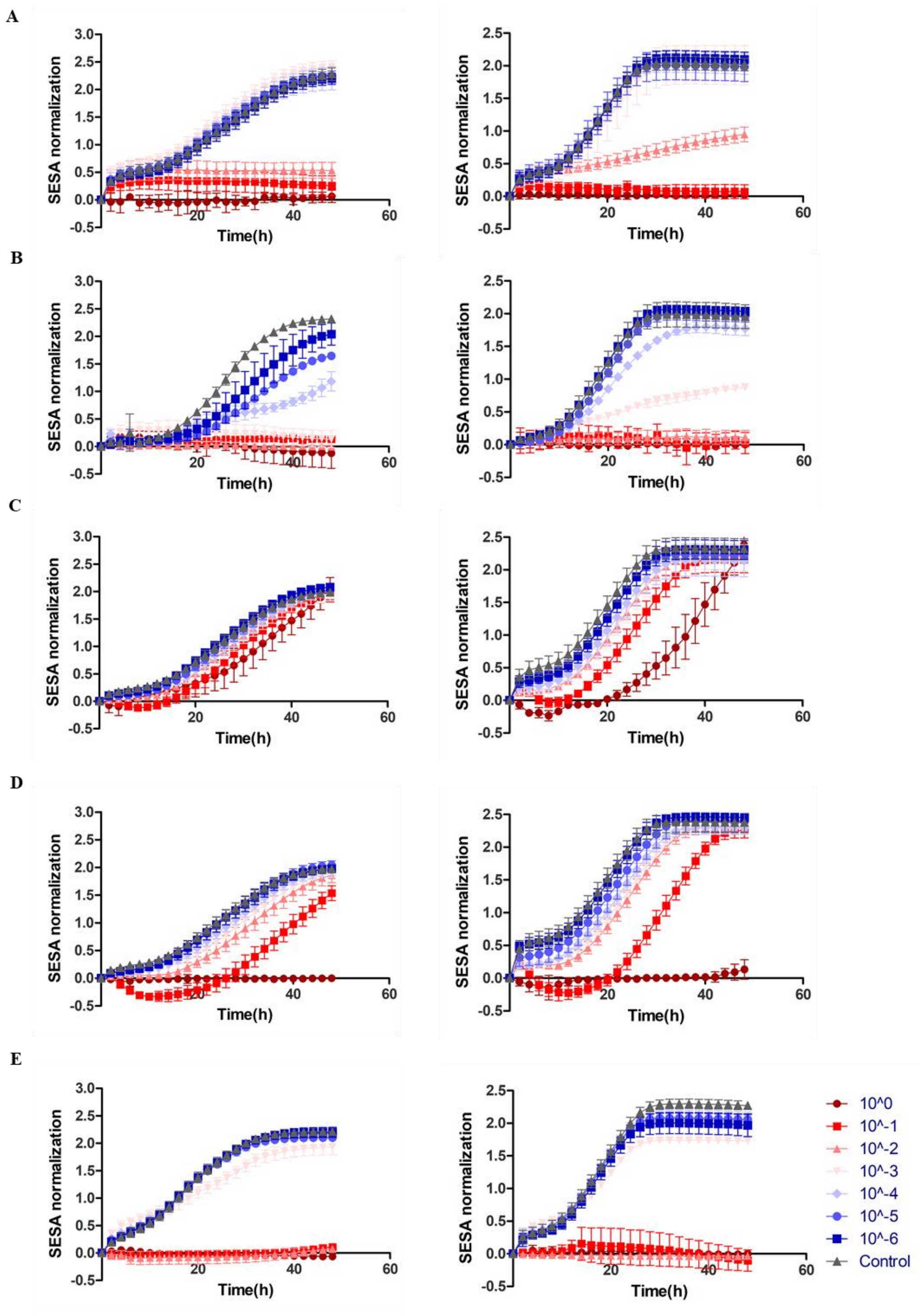

Supplementary Figure 4 Growth curves of *C. inversicolor* (left) and *P. roqueforti* (right) in the presence of targeted single VOCs (**(A)** acetic acid, **(B)** 3-methylbutanoic acid, **(C)** acetone, **(D)** 2-pentanone, and **(E)** 2-phenylethanol) at different concentrations (10^0, 10^{-1}, 10^{-2}, 10^{-3}, 10^{-4}, 10^{-5}, and 10^{-6} mol/L). Growth curves of mold species were measured as Normalized SESA units using oCelloScope^{TM}. Each spot is the mean value from three independent experiments, and the error bars represent standard deviation.
