# Supplementary material for: Debaryomyces hansenii Strains Isolated From Danish Cheese Brines Act as Biocontrol Agents to Inhibit Germination and Growth of Contaminating Molds
Source: Front Microbiol. 2021 Jun 15;12:662785. doi: 10.3389/fmicb.2021.662785 (PMC8239395; doi:10.3389/fmicb.2021.662785)
Supplement: Supplementary file 5 [file Table_1.DOCX]

**Supplementary Table 1** Estimated sizes of chromosomal bands (PFGE) for *D. hansenii* strains isolated from Danish dairies

| **PFGE Cluster** | **Strain** | **Size of chromosomal band (Mb)** | | | | |  |  |
| --- | --- | --- | --- | --- | --- | --- | --- | --- |
|  |  | **1** | **2** | **3** | **4** | **5** | **6** | **7** |
| Ⅰ | KU-78 | 3.14 | 2.66 | 2.50 | 2.03 | 1.80 | 1.49 |  |
| Ⅰ | KU-80 | 3.14 | 2.48 | 2.14 | 2.01 | 1.94 | 1.79 | 1.69 |
| Ⅱ | KU-12 | 3.14 | 2.13 | 1.95 | 1.79 | 1.03 |  |  |
| Ⅱ | KU-30 | 3.13 | 2.39 | 2.12 | 1.97 | 1.79 | 1.65 | 1.05 |
| Ⅱ | KU-9 | 3.14 | 2.17 | 1.82 | 1.54 | 1.04 |  |  |
| Ⅱ | KU-72 | 3.13 | 2.45 | 2.12 | 1.81 | 1.42 | 0.98 |  |
| Ⅲ | KU-11 | 3.14 | 2.17 | 1.85 | 1.46 | 0.95 |  |  |
| Ⅲ | KU-28 | 3.14 | 2.15 | 1.79 | 1.47 | 1.04 |  |  |
| Ⅳ | KU-10 | 3.13 | 2.57 | 2.46 | 2.29 | 2.08 | 1.95 | 1.86 |
| Ⅳ | KU-29 | 3.14 | 2.55 | 2.23 | 2.08 | 1.92 | 1.80 |  |
| Ⅳ | KU-27 | 3.14 | 2.52 | 2.29 | 1.98 | 1.81 |  |  |
|  | *D. hansenii* 767^*^ | 2.31 | 2.05 | 2.01 | 1.61 | 1.59 | 1.34 | 1.25 |

* The information for *D. hansenii* CDB767^T^ is obtained from NCBI (*Debaryomyces hansenii* (ID 195) - Genome - NCBI).

Clusters are based on chromosome polymorphism between the stains as determined by PFGE.
