# Supplementary material for: Debaryomyces hansenii Strains Isolated From Danish Cheese Brines Act as Biocontrol Agents to Inhibit Germination and Growth of Contaminating Molds
Source: Front Microbiol. 2021 Jun 15;12:662785. doi: 10.3389/fmicb.2021.662785 (PMC8239395; doi:10.3389/fmicb.2021.662785)
Supplement: Supplementary file 6 [file Table_2.DOCX]

| **Supplementary Table 2** VOCs identified in MYGP broth added 4% (w/v) NaCl inoculated with different *D. hansenii* strains for 72h at 25 °C | | | | | | | | | | | | | | | | | | | |
| --- | --- | --- | --- | --- | --- | --- | --- | --- | --- | --- | --- | --- | --- | --- | --- | --- | --- | --- | --- |
|  | **VOCs** | **MW** | **MF** | **RI_exp_^a^** | **RI_auth.sd_^b^** | **RI_lit_^c^** | **Relative peak area of volatile compounds * 10^ef^** | | | | | | | | | | | | |
|  |  |  |  |  |  |  | **Blank** | **KU-9** | **KU-10** | **KU-11** | **KU-12** | **KU-27** | **KU-28** | **KU-29** | **KU-30** | **KU-72** | **KU-78** | **KU-80** | **Identification^d^** |
| 1 | Acetone | 58 | C_3_H_6_O | 813 |  | 820 | 0.32^AB^ | 0.14^A^ | 0.36^ABC^ | 0.70^ABC^ | 1.03^ABC^ | 2.13^CD^ | 0.19^A^ | 0.01^A^ | 1.09^ABC^ | 2.06^BCD^ | 1.61^ABCD^ | 2.9^D^ | MS, KI |
| 2 | 2-methyl-propanal | 72 | C_4_H_8_O | 811 | 812 |  | 1.53^AB^ | 1.05^AB^ | 2.65^AB^ | 6.20^BCD^ | 5.35^ABC^ | 8.21^CD^ | 1.79^AB^ | 0.55^A^ | 4.60^ABC^ | 4.12^ABC^ | 10.44^D^ | 30.46^E^ | MS, STD |
| 3 | Butanal | 72 | C_4_H_8_O | 873 |  | 876 | 7.76^ABC^ | 2.67^AB^ | 6.47^ABC^ | 5.43^AB^ | 5.63^AB^ | 15.61^D^ | 8.09^BC^ | 6.04^ABC^ | 3.16^AB^ | 2.91^AB^ | 1.25^A^ | 12.22^CD^ | MS, KI |
| 4 | Ethyl acetate | 88 | C_4_H_8_O_2_ | 890 |  | 887 | 12.76^AB^ | 19.03^AB^ | 38.69^BC^ | 7.48^A^ | 17.22^AB^ | 83.15^D^ | 19.06^AB^ | 49.42^C^ | 14.87^AB^ | 8.33^A^ | 41.25^BC^ | 33.73^ABC^ | MS, KI |
| 5 | 2-Butanone | 72 | C_4_H_8_O | 903 | 906 |  | 3.46^F^ | 0.69^A^ | 1.23^ABC^ | 2.01^CDE^ | 2.73^EF^ | 1.97^CDE^ | 0.79^A^ | 1.10^AB^ | 2.40^DE^ | 2.43^DE^ | 1.67^BCD^ | 3.33^F^ | MS, STD |
| 6 | 2-methyl-butanal | 86 | C_5_H_10_O | 910 | 912 |  | 13.73^AB^ | 7.55^A^ | 14.26^ABC^ | 10.45^A^ | 11.34^A^ | 24.21^C^ | 9.22^A^ | 5.70^A^ | 8.23^A^ | 8.24^A^ | 22.92^BC^ | 68.50^D^ | MS, STD |
| 7 | 3-methyl-butanal | 86 | C_5_H_10_O | 915 | 916 |  | 41.08^C^ | 5.35^A^ | 8.73^AB^ | 8.07^AB^ | 7.49^AB^ | 9.72^AB^ | 7.12^AB^ | 2.63^A^ | 5.35^A^ | 8.73^AB^ | 2.18^B^ | 82.16^D^ | MS, STD |
| 8 | Ethanol | 46 | C_2_H_6_O | 938 |  | 929 | 0.03^A^ | 2.72^B^ | 2.38^B^ | 2.28^B^ | 2.46^B^ | 6.70^C^ | 2.82^B^ | 2.16^B^ | 2.99^B^ | 2.90^B^ | 1.21^AB^ | 3.2^8B^ | MS, KI |
| 9 | Ethyl propionate | 102 | C_5_H_10_O_2_ | 954 | 956 |  | 0.11^A^ | 2.40^B^ | 1.03^AB^ | 0.41^AB^ | 1.17^ABC^ | 3.80^E^ | 1.73^BCD^ | 2.74^DE^ | 1.30^ABC^ | 0.45^AB^ | 0.22^A^ | 0.82^AB^ | MS, STD |
| 10 | Ethyl isobutyrate | 116 | C_6_H_12_O_2_ | 964 | 968 |  | 0.03^A^ | 1.38^E^ | 0.12^AB^ | 0.66^BCD^ | 0.56^ABCD^ | 0.31^ABC^ | 0.98^DE^ | 0.21^ABC^ | 0.76^CD^ | 0.23^ABC^ | 0.08^AB^ | 0.19^ABC^ | MS, STD |
| 11 | 2-pentanone | 86 | C_5_H_10_O | 972 | 977 |  | 0.86^A^ | 0.89^A^ | 1.30^AB^ | 1.08^AB^ | 1.67^AB^ | 3.22^C^ | 1.04^A^ | 1.79^AB^ | 1.38^AB^ | 1.57^AB^ | 1.22^AB^ | 2.13^B^ | MS, STD |
| 12 | 2,3-butanedione | 86 | C_4_H_6_O_2_ | 977 | 985 |  | 1.57^A^ | 1.27^A^ | 0.90^A^ | 0.96^A^ | 4.20^C^ | 1.46^A^ | 1.53^A^ | 0.56^A^ | 5.34^D^ | 3.02^B^ | 0.72^A^ | 1.27^A^ | MS, STD |
| 13 | Methyl isobutyl ketone | 100 | C_6_H_12_O | 1007 |  | 1014 | 0.35^A^ | 0.36^A^ | 0.46^A^ | 0.41^AB^ | 0.50^A^ | 2.51^B^ | 0.55^A^ | 0.52^A^ | 0.52^A^ | 0.36^A^ | 0.68^A^ | 0.93^A^ | MS, KI |
| 14 | Isobutyl acetate | 116 | C_6_H_12_O_2_ | 1016 | 1018 |  | 0.03^A^ | 0.36^A^ | 1.71^B^ | 0.04^A^ | 0.12^A^ | 3.14^C^ | 0.26^A^ | 2.80^C^ | 0.16^A^ | ND | 1.87^B^ | 0.79^A^ | MS, STD |
| 15 | Ethyl butanoate | 116 | C_6_H_12_O_2_ | 1041 | 1040 |  | 0.05^A^ | 29.26^D^ | 0.89^A^ | 5.17^AB^ | 16.02^BC^ | 1.83^A^ | 16.10^BC^ | 1.94^A^ | 17.24^C^ | 6.16^ABC^ | 0.56^A^ | 0.98^A^ | MS, STD |
| 16 | 1-propanol | 60 | C_3_H_8_O | 1053 | 1048 |  | ND | 9.01^A^ | 23.06^A^ | 5.27^A^ | 11.17^A^ | 99.68^B^ | 10.67^A^ | 18.19^A^ | 24.96^A^ | 4.55^A^ | 5.27^A^ | 11.17^A^ | MS, STD |
| 17 | 2,3-pentanedione | 100 | C_5_H_8_O_2_ | 1070 | 1073 |  | 0.86^F^ | 0.05^AB^ | 0.03^AB^ | 0.02^A^ | 0.12^D^ | 0.05^AB^ | 0.05^AB^ | 0.04^AB^ | 0.15^E^ | 0.09^CD^ | 0.04^AB^ | 0.07^BC^ | MS, STD |
| 18 | Butyl acetate | 116 | C_6_H_12_O_2_ | 1081 | 1082 |  | 0.07^A^ | 0.88^B^ | 0.43^AB^ | 0.04^A^ | 0.10^A^ | 0.88^B^ | 0.57^AB^ | 1.00^B^ | 0.07^A^ | 0.02^A^ | 0.03^A^ | 0.08^A^ | MS, STD |
| 19 | 2-hexanone | 100 | C_6_H_12_O | 1085 |  | 1086 | 0.16^A^ | 0.15^A^ | 0.14^A^ | 0.15^A^ | 0.17^A^ | 0.29^B^ | 0.16^A^ | 0.17^A^ | 0.15^A^ | 0.13^A^ | 0.19^A^ | 0.22^AB^ | MS, KI |
| 20 | Hexanal | 100 | C_6_H_12_O | 1086 | 1087 |  | 0.85^C^ | 0.31^AB^ | 0.32^AB^ | 0.32^AB^ | 0.38^AB^ | 0.70^BC^ | 0.29^AB^ | 0.18^A^ | 0.44^AB^ | 0.22^A^ | 0.49^ABC^ | 0.59^ABC^ | MS, STD |
| 21 | 2-methyl-(E)-2-butenal | 84 | C_5_H_8_O | 1091 | 1092 |  | 0.75^B^ | 0.08^A^ | 0.06^A^ | 0.04^A^ | 0.03^A^ | 0.03^A^ | 0.06^A^ | 0.03^A^ | 0.02^A^ | 0.01^A^ | 0.02^A^ | 0.07^A^ | MS, STD |
| 22 | 2-methyl-1-Propanol | 74 | C_4_H_10_O | 1111 | 1110 |  | 1.96^A^ | 225.20^BC^ | 286.09^BC^ | 168.85^AB^ | 336.73^BC^ | 594.33^D^ | 299.96^BC^ | 284.85^BC^ | 380.75^C^ | 236.00^BC^ | 369.27^C^ | 358.31^BC^ | MS, STD |
| 23 | 3-methyl-1-Butanol acetate | 130 | C_7_H_14_O_2_ | 1132 | 1142 |  | 0.22^A^ | 1.39^BC^ | 1.71^CD^ | 0.18^A^ | 0.47^A^ | 2.61^E^ | 0.77^AB^ | 2.47^DE^ | 0.58^AB^ | 0.05^A^ | 1.86^CDE^ | 1.33^BC^ | MS, STD |
| 24 | 4-methyl-3-penten-2-one | 98 | C_6_H_10_O | 1136 |  | 1127 | 0.98^A^ | 0.20^A^ | 0.10^A^ | 0.14^A^ | 0.15^A^ | 3.30^B^ | 0.59^A^ | 0.19^A^ | 0.16^A^ | 0.09^A^ | 0.12^A^ | 0.38^A^ | MS, KI |
| 25 | 2-Pentanol | 88 | C_5_H_12_O | 1141 |  | 1118 | 0.00^A^ | 0.45^AB^ | 0.40^AB^ | 0.38^AB^ | 0.40^AB^ | 1.56^C^ | 0.57^B^ | 0.42^AB^ | 0.51^B^ | 0.36^AB^ | 0.39^AB^ | 0.42^AB^ | MS, KI |
| 26 | 1-Butanol | 74 | C_4_H_10_O | 1173 | 1165 |  | 566.09^DE^ | 310.41^BC^ | 365.14^BCD^ | 182.23^AB^ | 290.01^BC^ | 642.1^0E^ | 493.15^BCD^ | 484.74^CDE^ | 204.09^AB^ | 152.25^AB^ | 53.80^A^ | 224.50^AB^ | MS, STD |
| 27 | 3-methyl-2-Butenal | 84 | C_5_H_8_O | 1201 |  | 1214 | 0.63^D^ | 0.04^ABC^ | 0.06^C^ | 0.03^AB^ | 0.03^AB^ | 0.05^BC^ | 0.03^AB^ | 0.05^BC^ | 0.01^A^ | 0.01^A^ | 0.02^A^ | 0.02^A^ | MS, KI |
| 28 | 3-methyl-1-Butanol | 88 | C_5_H_12_O | 1238 | 1238 |  | 5.36^A^ | 533.00^BC^ | 486.70^B^ | 498.39^B^ | 663.25^BC^ | 751.92^CD^ | 630.71^BC^ | 519.93^B^ | 681.85^BCD^ | 689.26^BCD^ | 751.00^CD^ | 896.64^D^ | MS, STD |
| 29 | Thiazole | 85 | C_3_H_3_NS | 1260 |  | 1249 | 0.78^C^ | 0.62^AB^ | 0.65^AB^ | 0.60^AB^ | 0.68^BC^ | 0.66^AB^ | 0.66^AB^ | 0.68^BC^ | 0.64^AB^ | 0.67^AB^ | 0.62^AB^ | 0.59^AB^ | MS, KI |
| 30 | Styrene | 104 | C_8_H_8_ | 1267 |  | 1260 | 0.05^A^ | 0.16^AB^ | 0.10^AB^ | 0.10^AB^ | 0.09^AB^ | 0.18^B^ | 0.21^B^ | 0.09^AB^ | 0.10^AB^ | 0.10^AB^ | 0.11^AB^ | 0.11^AB^ | MS, KI |
| 31 | 3-methyl-3-buten-1-ol | 86 | C_5_H_10_O | 1267 |  | 1249 | 0.02^A^ | 0.66^B^ | 1.02^CD^ | 1.09^CD^ | 1.03^CD^ | 1.08^CD^ | 0.62^B^ | 1.22^CDE^ | 1.02^CD^ | 1.49^E^ | 0.88^BC^ | 1.35^DE^ | MS, KI |
| 32 | 1-pentanol | 88 | C_5_H_12_O | 1271 | 1273 |  | 0.27^A^ | 1.28^DE^ | 1.50^BCD^ | 0.89^BC^ | 1.22^CDE^ | 1.41^E^ | 1.24^DE^ | 1.13^BCDE^ | 1.10^BCDE^ | 0.90^BC^ | 0.87^B^ | 1.18^BCDE^ | MS, STD |
| 33 | Dihydro-2-methyl-3(2H)-furanone | 100 | C_5_H_8_O_2_ | 1275 |  | 1267 | 0.23^D^ | 0.22^CD^ | 0.17^ABC^ | 0.14^AB^ | 0.20^BCD^ | 0.32^E^ | 0.22^CD^ | 0.22^CD^ | 0.20^CD^ | 0.16^ABC^ | 0.13^A^ | 0.17^ABC^ | MS, KI |
| 34 | Acetoin | 88 | C_4_H_8_O_2_ | 1297 | 1307 |  | 0.29^A^ | 7.67^B^ | 4.94^AB^ | 4.26^AB^ | 26.21^D^ | 4.61^AB^ | 6.48^AB^ | 3.93^AB^ | 22.70^D^ | 14.63^C^ | 3.10^AB^ | 6.68^AB^ | MS, STD |
| 35 | Octanal | 128 | C_8_H_16_O | 1304 | 1306 |  | 0.63^A^ | 0.71^AB^ | 0.53^A^ | 0.53^A^ | 0.51^A^ | 0.92^B^ | 0.57^A^ | 0.44^A^ | 0.58^A^ | 0.43^A^ | 0.60^A^ | 0.72^AB^ | MS, STD |
| 36 | 1-hydroxy-2-propanone | 74 | C_3_H_6_O_2_ | 1311 |  | 1301 | 0.10^BC^ | 0.09^AB^ | 0.07^AB^ | 0.07^AB^ | 0.17^C^ | 0.07^AB^ | 0.06^AB^ | 0.03^AB^ | 0.09^AB^ | 0.04^AB^ | 0.03^AB^ | 0.02^A^ | MS, KI |
| 37 | 4-penten-1-ol | 86 | C_5_H_10_O | 1319 |  | 1296 | ND | 0.32^DE^ | 0.30^DE^ | 0.20^C^ | 0.27^D^ | 0.34^E^ | 0.26^D^ | 0.44^F^ | 0.28^DE^ | 0.18^BC^ | 0.03^A^ | 0.13^B^ | MS, KI |
| 38 | 2-methyl-1-pentanol | 102 | C_6_H_14_O | 1319 |  | 1294 | ND | 0.39^BC^ | 0.36^BC^ | 0.23^B^ | 0.44^C^ | 0.52^C^ | 0.36^BC^ | 0.37^BC^ | 0.38^BC^ | 0.23^B^ | 0.37^BC^ | 0.39^BC^ | MS, KI |
| 39 | 3-methyl-3-phenyl-azetidine | 147 | C_10_H_13_N | 1343 |  |  | 0.09^BC^ | 0.17^D^ | 0.06^AB^ | 0.09^BC^ | 0.10^BC^ | 0.12^C^ | 0.08^BC^ | 0.03^A^ | 0.03^A^ | 0.03^A^ | 0.02^A^ | 0.02^A^ | MS |
| 40 | 3-methyl-1-pentanol | 102 | C_6_H_14_O | 1345 |  | 1326 | 0.14^A^ | 0.79^C^ | 0.83^C^ | 0.06^B^ | 0.79^C^ | 1.09^D^ | 0.80^C^ | 0.90^C^ | 0.77^C^ | 0.56^B^ | 0.79^C^ | 0.87^C^ | MS, KI |
| 41 | 1-hexanol | 102 | C_6_H_14_O | 1368 | 1371 |  | 0.25^A^ | 0.60^BC^ | 0.31^A^ | 0.38^AB^ | 0.43^AB^ | 0.41^AB^ | 0.71^C^ | 0.38^AB^ | 0.43^AB^ | 0.41^AB^ | 0.24^A^ | 0.30^A^ | MS, STD |
| 42 | Nonanal | 142 | C_9_H_18_O | 1402 | 1402 |  | 0.58^A^ | 1.01^CDE^ | 0.72^ABC^ | 0.96^BCDE^ | 1.10^DE^ | 1.50^F^ | 0.82^ABCD^ | 0.75^ABC^ | 1.26^EF^ | 0.66^AB^ | 1.21^EF^ | 1.25^EF^ | MS, STD |
| 43 | 1,3-bis(1,1-dimethylethyl)-benzene | 190 | C_14_H_22_ | 1435 |  |  | ND | 0.29^B^ | 0.23^AB^ | 0.39^B^ | 0.24^B^ | 0.39^B^ | 0.16^AB^ | 0.22^AB^ | 0.34^B^ | 0.20^AB^ | 0.37^B^ | 0.34^B^ | MS |
| 44 | Acetic acid | 60 | C_2_H_4_O_2_ | 1453 |  | 1447 | 0.12^A^ | 0.15^A^ | 0.23^AB^ | 0.25^A^ | 0.40^A^ | 0.56^A^ | 0.21^A^ | 0.21^A^ | 0.21^A^ | 0.43^B^ | 0.30^A^ | 0.23^A^ | MS, KI |
| 45 | Methional | 104 | C_4_H_8_OS | 1463 | 1467 |  | 0.49^B^ | 0.01^A^ | 0.00^A^ | 0.00^A^ | 0.00^A^ | ND | 0.00^A^ | 0.00^A^ | 0.00^A^ | 0.01^A^ | ND | 0.01^A^ | MS, STD |
| 46 | Furfural | 96 | C_5_H_4_O_2_ | 1471 |  | 1461 | 19.21^B^ | 0.43^A^ | 0.47^A^ | 0.41^A^ | 0.49^A^ | 0.94^A^ | 0.47^A^ | 0.41^A^ | 0.51^A^ | 0.48^A^ | 0.58^A^ | 0.74^A^ | MS, KI |
| 47 | 2-ethyl-1-hexanol | 130 | C_8_H_18_O | 1499 | 1499 |  | 0.33^A^ | 2.06^B^ | 1.94^B^ | 2.05^B^ | 2.25^B^ | 2.48^BC^ | 2.61^BC^ | 3.05^BC^ | 3.00^BC^ | 2.05^B^ | 3.07^BC^ | 3.38C | MS, STD |
| 48 | Formic acid | 46 | CH_2_O_2_ | 1509 |  | 1504 | 0.04^A^ | 0.06^A^ | 0.15^AB^ | 0.11^A^ | 0.25^B^ | 0.17^AB^ | 0.06^A^ | 0.09^AB^ | 0.22^B^ | 0.07^A^ | 0.08^A^ | 0.05^A^ | MS, KI |
| 49 | Benzaldehyde | 106 | C_7_H_6_O | 1534 | 1531 |  | 6.91^G^ | 0.65^AB^ | 0.53^AB^ | 1.29^C^ | 1.87^E^ | 0.54^AB^ | 0.81^B^ | 0.26^A^ | 1.49^CD^ | 2.31^F^ | 0.54^AB^ | 1.72^DE^ | MS, STD |
| 50 | 1-octanol | 130 | C_8_H_18_O | 1569 | 1570 |  | 0.25^BC^ | 0.19^AB^ | 0.14^A^ | 0.16^A^ | 0.18^AB^ | 0.25^BC^ | 0.13^A^ | 0.13^A^ | 0.20^AB^ | 0.14^A^ | 0.19^AB^ | 0.28^C^ | MS, STD |
| 51 | 1-(2,4-Dimethyl-furan-3-yl)-ethanone | 138 | C_8_H_10_O_2_ | 1587 |  |  | 0.30^A^ | 0.04^ABC^ | 0.02^A^ | 0.05^A^ | 0.02^A^ | 1.87^B^ | 0.19^A^ | 0.07^A^ | 0.03^A^ | 0.02^A^ | 0.05^A^ | 0.05^A^ | MS |
| 52 | Propylene glycol | 76 | C_3_H_8_O_2_ | 1602 |  | 1600 | 0.14^ABC^ | 0.15^ABC^ | 0.17^ABC^ | 0.22^C^ | 0.20^BC^ | 0.05^A^ | 0.04^A^ | 0.06^AB^ | 0.06^AB^ | 0.02^A^ | 0.03^A^ | 0.03^A^ | MS, KI |
| 53 | Benzeneacetaldehyde | 120 | C_8_H_8_O | 1653 | 1659 |  | 3.91^E^ | 0.13^B^ | 0.13^B^ | 0.07^AB^ | 0.10^AB^ | 0.08^AB^ | 0.08^AB^ | 0.06^A^ | 0.08^AB^ | 0.09^AB^ | 0.24^C^ | 0.50^D^ | MS |
| 54 | 2-furanmethanol | 98 | C_5_H_6_O_2_ | 1667 | 1679 |  | 0.26^A^ | 0.38^AB^ | 0.33^AB^ | 0.33^AB^ | 0.36^AB^ | 0.31^AB^ | 0.39^AB^ | 0.37^AB^ | 0.36^AB^ | 0.40^B^ | 0.25^A^ | 0.32^AB^ | MS |
| 55 | Acetophenone | 120 | C_8_H_8_O | 1666 |  | 1646 | 0.15^E^ | 0.05^AB^ | 0.05^AB^ | 0.08^BCD^ | 0.10^CD^ | 0.11^D^ | 0.03^A^ | 0.04^AB^ | 0.08^BCD^ | 0.07^ABCD^ | 0.06^ABC^ | 0.08^BCD^ | MS, KI |
| 56 | 3-methyl-butanoic acid | 116 | C_6_H_12_O_2_ | 1673 |  | 1680 | 0.05^AB^ | 0.03^A^ | 0.04^A^ | 0.08^AB^ | 0.09^AB^ | 0.07^AB^ | 0.06^AB^ | 0.08^AB^ | 0.11^AB^ | 0.07^AB^ | 0.22^B^ | 0.61^C^ | MS, KI |
| 57 | 3-thiophenecarboxaldehyde | 112 | C_5_H_4_OS | 1692 |  | 1679 | 0.06 | ND | ND | ND | ND | ND | ND | ND | ND | ND | ND | ND | MS, KI |
| 58 | 3-(methylthio)-1-propanol | 106 | C_4_H_4_OS | 1731 |  | 1720 | 0.00^A^ | 0.27^EF^ | 0.14^BC^ | 0.13^BC^ | 0.17^BC^ | 0.10^BC^ | 0.24^DE^ | 0.33^F^ | 0.17^CD^ | 0.12^BC^ | 0.09^ABC^ | 0.07^AB^ | MS, KI |
| 59 | à,à-dimethyl-benzenemethanol | 136 | C_9_H_12_O | 1771 |  |  | 0.17^ABC^ | 0.28^CD^ | 0.15^ABC^ | 0.32^D^ | 0.26^BCD^ | 0.23^BCD^ | 0.18^ABC^ | 0.12^AB^ | 0.12^AB^ | 0.12^AB^ | 0.08^A^ | 0.14^ABC^ | MS |
| 60 | 2,5-dimethyl-benzaldehyde | 134 | C_9_H_10_O | 1836 |  |  | 1.32^B^ | 0.21^A^ | 0.20^A^ | 0.20^A^ | 0.23^A^ | 0.19^A^ | 0.26^A^ | 0.19^A^ | 0.29^A^ | 0.21^A^ | 0.26^A^ | 0.31^A^ | MS |
| 61 | 1,1'-oxybis-2-propanol | 134 | C_6_H_14_O_3_ | 1845 |  |  | 0.04^A^ | 0.05^A^ | 0.04^A^ | 0.21^B^ | 0.26^B^ | 0.01^A^ | 0.01^A^ | 0.02^A^ | 0.03^A^ | 0.01^A^ | 0.01^A^ | 0.06^A^ | MS |
| 62 | 3-phenyl-furan | 144 | C_10_H_8_O | 1869 |  | 1851 | 0.20^D^ | 0.08^C^ | 0.04^ABC^ | 0.04^ABC^ | 0.02^A^ | 0.07^BC^ | 0.05^ABC^ | 0.04^ABC^ | 0.03^AB^ | 0.02^A^ | 0.02^A^ | 0.02^A^ | MS, KI |
| 63 | 3,3'-oxybis-1-propanol | 134 | C_6_H_14_O_3_ | 1904 |  |  | 0.02^A^ | 0.02^A^ | 0.02^A^ | 0.15^B^ | 0.18^B^ | 0.01^A^ | 0.01^A^ | 0.01^A^ | 0.01^A^ | 0.01^A^ | 0.01^A^ | 0.01^A^ | MS |
| 64 | 2‐phenylethanol | 122 | C_8_H_10_O | 1928 | 1930 |  | 0.01^A^ | 3.72^CD^ | 3.38^BCD^ | 4.72^CD^ | 3.34^BCD^ | 1.99^B^ | 3.51^BCD^ | 3.08^BC^ | 3.12^BC^ | 4.84^D^ | 3.34^BCD^ | 3.40^BCD^ | MS, STD |
| 65 | Phenol | 94 | C_6_H_6_O | 2010 |  | 1997 | 0.05^A^ | 0.06^A^ | 0.09^A^ | 0.09^A^ | 0.11^A^ | 0.19^B^ | 0.07^A^ | 0.06^A^ | 0.12^AB^ | 0.09^A^ | 0.10^A^ | 0.10^A^ | MS, KI |
| 66 | Octanoic acid | 144 | C_8_H_16_O_2_ | 2065 |  | 2060 | 0.06^AB^ | 0.06^AB^ | 0.08^AB^ | 0.10^AB^ | 0.24^B^ | 0.08^AB^ | 0.05^AB^ | 0.03^A^ | 0.08^AB^ | 0.03^A^ | 0.04^AB^ | 0.01^A^ | MS, KI |
| 67 | Nonanoic acid | 158 | C_9_H_18_O_2_ | 2169 |  | 2167 | 0.08^A^ | 0.08^A^ | 0.13^AB^ | 0.14^AB^ | 0.16^B^ | 0.16^B^ | 0.07^A^ | 0.07^A^ | 0.13^AB^ | 0.07^A^ | 0.08^A^ | 0.03^A^ | MS, KI |
| 68 | 3-decoxythiolane 1,1-dioxide | 120 | C_4_H_8_O_2_S | 2256 |  |  | 0.07^A^ | 0.09^AB^ | 0.08^AB^ | 0.09^AB^ | 0.11^AB^ | 0.14^B^ | 0.08^AB^ | 0.07^A^ | 0.09^AB^ | 0.06^A^ | 0.06^A^ | 0.09^AB^ | MS |
| 69 | n-decanoic acid | 172 | C_10_H_20_O_2_ | 2263 |  | 2276 | 0.09^A^ | 0.11^A^ | 0.19^A^ | 0.18^A^ | 0.39^C^ | 0.24^BC^ | 0.12^A^ | 0.10^A^ | 0.16^A^ | 0.08^A^ | 0.08^A^ | 0.02^A^ | MS, KI |
| 70 | 2,4-bis(1,1-dimethylethyl)-phenol | 206 | C_14_H_22_O | 2289 |  | 2313 | 0.01^A^ | 0.09^AB^ | 0.02^A^ | 0.17^AB^ | 0.04^A^ | 0.02^A^ | 0.06^AB^ | 0.23^B^ | 0.03^A^ | 0.12^AB^ | 0.09^AB^ | 0.06^AB^ | MS, KI |
| 71 | Diethyl phthalate | 222 | C_12_H14O_4_ | 2354 |  | 2360 | 0.12 | 0.14 | 0.11 | 0.25 | 0.17 | 0.44 | 0.16 | 0.15 | 0.22 | 0.16 | 0.16 | 0.14 | MS, KI |

a RI _exp_Kovats indices calculated from retention time data on a polar column.

b RI _exp_Kovats indices from authentic standards.

c RI _lit_Kovats indices the mean of Kovats from PubChem database.

d Identification by comparison with MS mass spectra, KI Kovats indices from literature, STD authentic standards purchased from Sigma Aldrich (St. Louis, Missouri, United States).

e The relative amount of volatile compounds represents the mean of individual relative peak area compared with internal standard (4-methyl-1-Pentanol)* 10.

f Mean values marked by different uppercase letters within same compound are significantly different using one-way ANOVA with Duncan’ s test (≥ 95% confidence)
